# High‐dimensional analyses reveal a distinct role of T‐cell subsets in the immune microenvironment of gastric cancer

**DOI:** 10.1002/cti2.1127

**Published:** 2020-05-05

**Authors:** Minyu Wang, Yu‐Kuan Huang, Joseph CH Kong, Yu Sun, Daniela G Tantalo, Han Xian Aw Yeang, Le Ying, Feng Yan, Dakang Xu, Heloise Halse, Natasha Di Costanzo, Ian R Gordon, Catherine Mitchell, Laura K Mackay, Rita A Busuttil, Paul J Neeson, Alex Boussioutas

**Affiliations:** ^1^ Upper Gastrointestinal Translational Research Laboratory Peter MacCallum Cancer Centre Melbourne VIC Australia; ^2^ Sir Peter MacCallum Department of Oncology The University of Melbourne Melbourne VIC Australia; ^3^ Department of Medicine, Royal Melbourne Hospital The University of Melbourne Melbourne VIC Australia; ^4^ Cancer Immunology Research Peter MacCallum Cancer Centre Melbourne VIC Australia; ^5^ Statistical Consulting Centre School of Mathematics and Statistics The University of Melbourne Melbourne VIC Australia; ^6^ Centre for Innate Immunity and Infectious Diseases Hudson Institute of Medical Research Clayton VIC Australia; ^7^ Australian Centre for Blood Diseases Central Clinical School Monash University Melbourne VIC Australia; ^8^ Faculty of Medical Laboratory Science Ruijin Hospital School of Medicine Shanghai Jiao Tong University Shanghai China; ^9^ Department of Pathology Peter MacCallum Cancer Centre Melbourne VIC Australia; ^10^ Department of Microbiology and Immunology Peter Doherty Institute for Infection and Immunity University of Melbourne Melbourne VIC Australia; ^11^ Department of Pathology The University of Melbourne Melbourne VIC Australia

**Keywords:** CD4^+^FOXP3^+^ T cells, CD8^+^ T cells, gastric cancer, interferon‐gamma gene signature

## Abstract

**Objectives:**

To facilitate disease prognosis and improve precise immunotherapy of gastric cancer (GC) patients, a comprehensive study integrating immune cellular and molecular analyses on tumor tissues and peripheral blood was performed.

**Methods:**

The association of GC patients’ outcomes and the immune context of their tumors was explored using multiplex immunohistochemistry (mIHC) and transcriptome profiling. Potential immune dysfunction mechanism/s in the tumors on the systemic level was further examined using mass cytometry (CyTOF) in complementary peripheral blood from selected patients. GC cohorts with mIHC and gene expression profiling data were also used as validation cohorts.

**Results:**

Increased CD4^+^FOXP3^+^ T‐cell density in the GC tumor correlated with prolonged survival. Interestingly, CD4^+^FOXP3^+^ T cells had a close interaction with CD8^+^ T cells rather than tumor cells. High densities of CD4^+^FOXP3^+^ T cells and CD8^+^ T cells (High‐High) independently predicted prolonged patient survival. Furthermore, the interferon‐gamma (IFN‐γ) gene signature and PDL1 expression were up‐regulated in this group. Importantly, a subgroup of genomically stable (GS) tumors and tumors with chromosomal instability (CIN) within this High‐High group also had excellent survival. The High‐High GS/CIN tumors were coupled with increased frequencies of Tbet^+^CD4^+^ T cells and central memory CD4^+^ T cells in the peripheral blood.

**Conclusion:**

These novel findings identify the combination of CD8^+^ T cells and FOXP3^+^CD4^+^ T cells as a significant prognostic marker for GC patients, which also could potentially be targeted and applied in the combination therapy with immune checkpoint blockades in precision medicine.

## Graphical abstract

In this work, we show an increased CD4^+^FOXP3^+^ T cell density in the tumour core correlated with prolonged survival and CD4^+^FOXP3^+^ T cells clustered with CD8^+^ T cells rather than tumour cells. High density of CD4^+^FOXP3^+^ T cells and CD8^+^ T cells (High‐High) independently predicted prolonged patient survival. These High‐High tumours were coupled with an increased IFN‐γ response, antigen presentation, DCs differentiation and PDL1 upregulation in the local tumours, as well as enrichment of Tbet^+^ CD4^+^ T cells and central memory CD4^+^ T cells circulating in the peripheral blood.

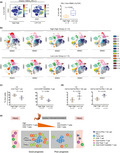

## Introduction

Gastric cancer (GC) is the fourth most common cancer and the second leading cause of cancer death worldwide.^1^ In 2014, The Cancer Genome Atlas (TCGA) subtyped GC into four molecular subtypes: tumors positive for Epstein–Barr virus (EBV), microsatellite unstable (MSI) tumors, genomically stable (GS) tumors and tumors with chromosomal instability (CIN).^2^ EBV‐positive tumors (9% of all GC) featured DNA hypermethylation, a high frequency of *PIK3CA* mutations and up‐regulation of *PDL1* and *PDL2* genes; MSI tumors (22%) showed an unusually high number of mutations and DNA methylation sites; CIN tumors (50%) harboured alterations in tyrosine kinase receptors; and GS tumors (20%) were characterised by *RHOA* mutations and are enriched for the diffuse histological type. Despite combination therapy with surgery and chemotherapy, the survival of patients with advanced GC has not changed significantly in many countries.^3^ The immune system has increasingly been recognised as a powerful tool in the treatment of cancer. Indeed, immune checkpoint blockade (ICB) has been successfully used to treat patients with a wide range of cancer types.^4^ However, objective response rates of ICB therapy in GC have been observed in only a subset of patients.^5^ This variability in response suggests that the tumor microenvironment is critical for patient selection for ICB and the development of targeted immunotherapy.

In colorectal cancer, the 'Immunoscore' reported that the location (core or invasive margin) of tumor‐infiltrating CD3^+^ T cells and CD8^+^ T cells correlated with long‐term survival of patients.^6^, ^7^ It is recognised that most of GC development occurs in the context of chronic inflammation induced by *Helicobacter pylori* ^8^ or Epstein–Barr virus.^9^ However, the assessment of T‐cell subsets as a prognostic biomarker in gastric cancer has led to controversial findings. Kim *et al.* reported that an increased number of CD8^+^ T cells was associated with improved survival in a Korean GC cohort.^10^, ^11^ However, the results in a Western cohort showed that an increased number of CD8^+^ T cells correlated with poor overall survival.^12^ In addition, the prognostic values of FOXP3^+^ T cells in the tumor were also controversial. Patients with a high number of FOXP3^+^ T cells in their tumor had a median survival time of 58 months, while those with a low FOXP3^+^ T cells count had a median survival time of 32 months.^13^ Kim *et al.* ^10^ reported a similar finding in a cohort of 99 MSI‐High GC patients showing a high density of FOXP3^+^ T cells was significantly associated with improved overall survival. However, a high number of intra‐tumoral FOXP3^+^ T cells significantly correlated with adverse survival in several studies.^11^, ^14^, ^15^, ^16^ These results highlight the need for further characterisation of the GC tumor immune microenvironment, which integrates both the cellular contexture and molecular expression to help elucidate the association between T‐cell subsets and clinical outcomes.

## Results

### Investigating the infiltration of immune cells in gastric tumors

**Figure 1:**
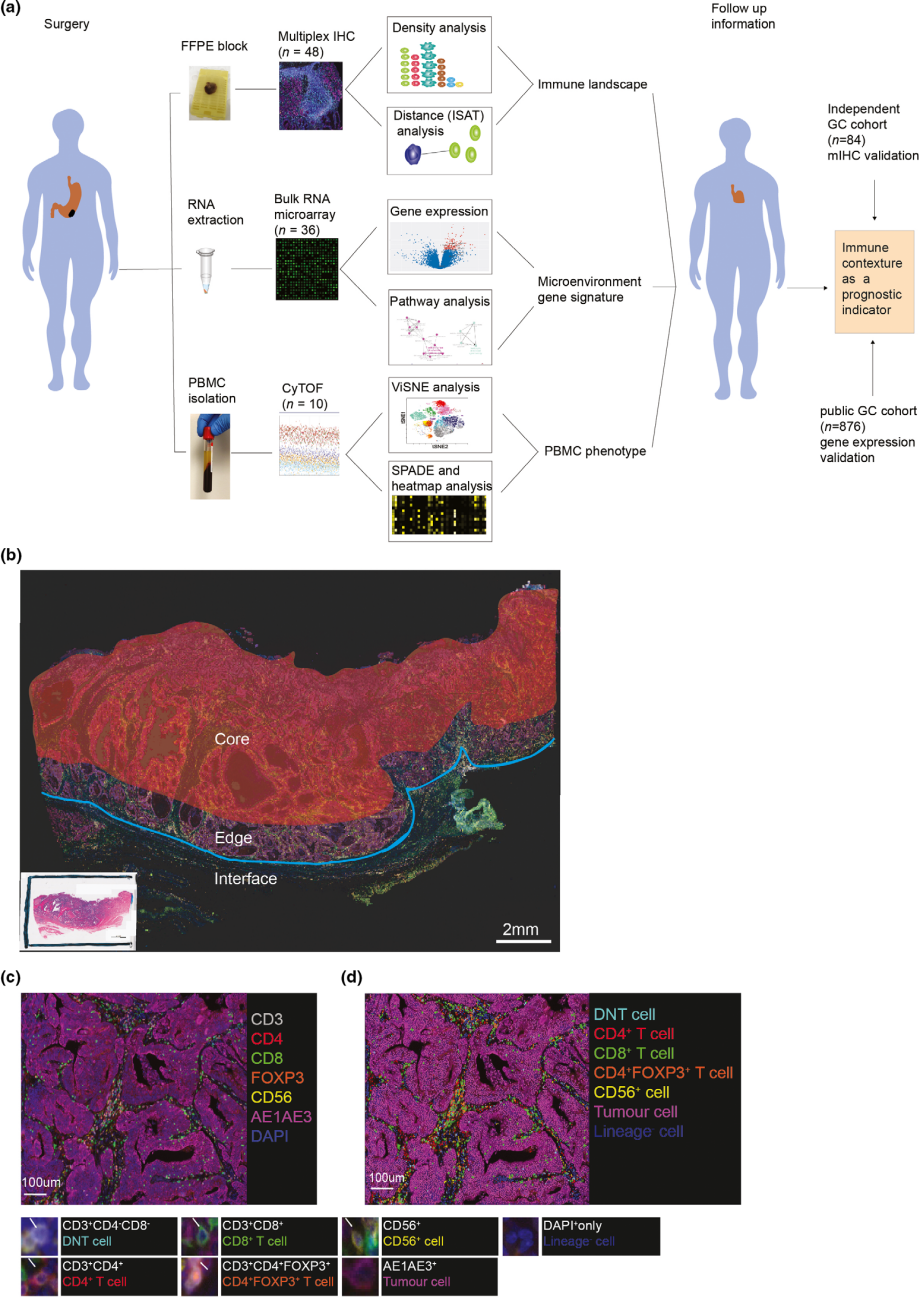
Using the high‐dimensional immune context data as a prognostic indicator in gastric cancer. **(a)** Overview of the study design. Gastric tumors were profiled using matched multiplex immunohistochemistry (mIHC, *n* = 48) and gene expression microarrays (*n* = 36). The systemic immunity for the patients was studied using mass cytometry (CyTOF) from selected patients (*n* = 10). The data were then correlated with clinical parameters and patient outcomes to develop a prognostic indicator, which was further validated using public GC cohorts by mIHC (*n* = 84) and gene expression data (*n* = 876). **(b)** H&E sections were used to identify regions for subsequent mIHC analysis where tumor ‘edge’ represent the area within 1mm of the interface between tumor and normal sites, and tumor ‘core’ represent the tumor area proximal to the tumor edge. **(c)** Representative composite image showing the distribution of individual markers (CD3, CD4, CD8, FOXP3, CD56, AE1AE3 and DAPI) within a tumor region. **(d)** The same image was phenotyped, allowing the identification of different immune cell subtypes (DNT cell, CD4^+^ T cell, CD8^+^ T cell, CD4^+^FOXP3^+^ T cell, CD56^+^ cell, tumor cell and lineage^−^ cell).

To comprehensively characterised the tumor‐infiltrating immune cells in GC tumors, we investigated different immune cell types, their densities, and spatial relationships, as well as matched molecular profiling data by integrating multiplex immunohistochemistry (mIHC), gene expression microarray and mass cytometry (CyTOF) analysis (Figure 1a). We first performed mIHC on surgical resection samples from 48 GC patients. Regions of interest were divided into tumor core and tumor edge based on the distance to the interface of tumor tissue and adjacent non‐tumor tissue by identifying the tumor cells with the positive staining of AE1AE3 (Figure 1b–d). The densities and spatial interactions of CD8^+^ T cells, CD4^+^ T cells (CD4^+^FOXP3^−^ T cells), CD4^+^FOXP3^+^ T cells, double‐negative T (DNT) cells, CD56^+^ cells (mainly nature killer cells), lineage^−^ cells (immune lineage negative, DAPI^+^) and tumor cells were studied (Figure 1c), and examples of stratified cell phenotypes are shown in Figure 1d.

**Figure 2:**
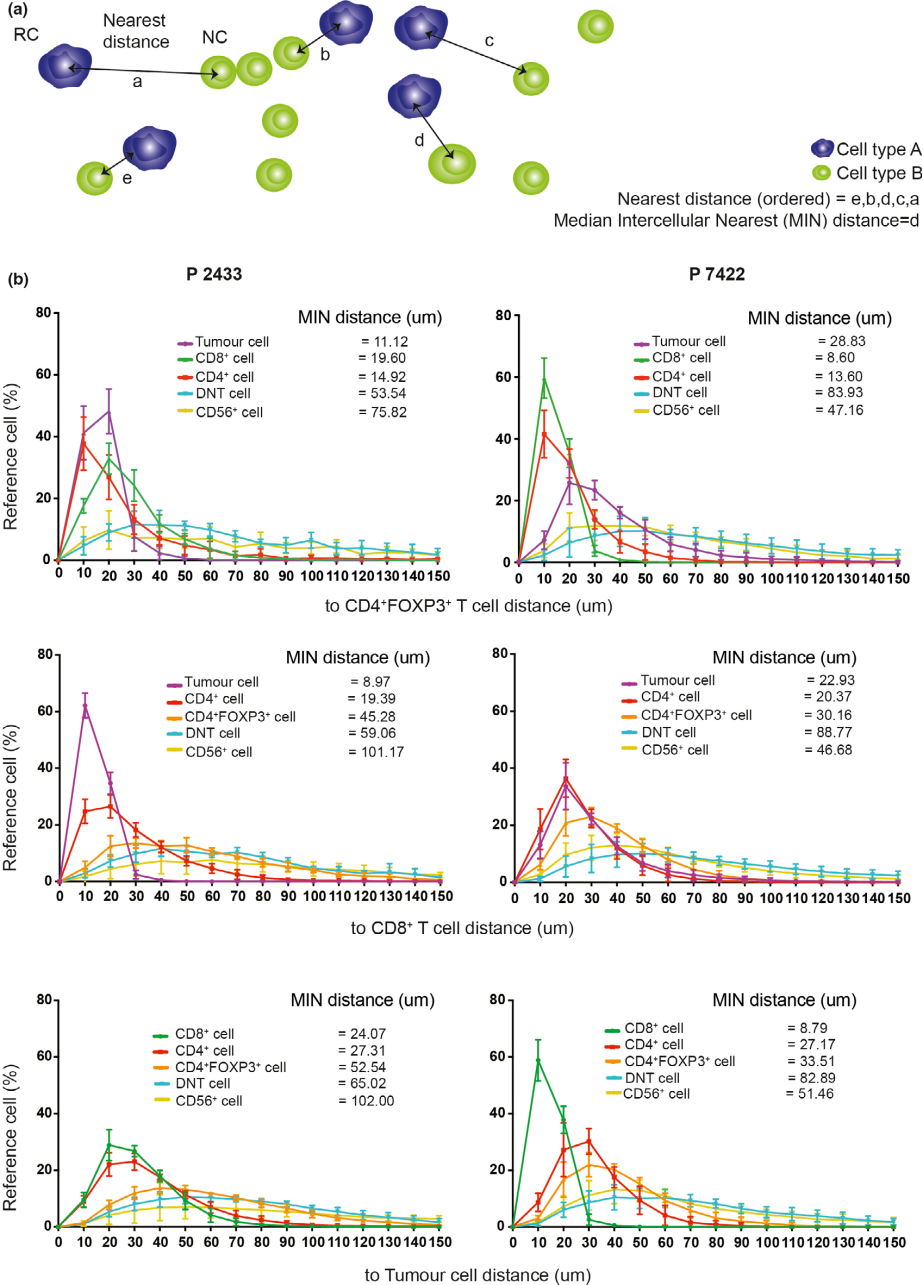
Gastric tumor samples display heterogeneous immune cells distribution. **(a)** Schematic representation of the rationale to calculate the intercellular nearest distance between the reference cell (RC) and nearest cell (NC). Median Intercellular Nearest (MIN) distance was defined as the median value of the nearest distances. **(b)** The coloured lines represent the percentage of RC within a certain nearest distance (range from 10 to 150 μm) between RC and NC. Shown are pairs of MIN distance analyses from P2433 and P7422 where the RC is CD4^+^FOXP3^+^ T cells, CD8^+^ T cells or Tumor cells. Data are presented as the mean ± SD.

We hypothesised that spatial relationships between individual cellular components in the tumor microenvironment might offer novel insights into the complex functions of tumor‐infiltrating immune cells. To investigate this, we established a novel computational method ‘Intercellular Spatial Analysis Tool (ISAT)’ to probe the spatial features of immune cells and tumor cells in the GC tumor microenvironment. In ISAT, ‘nearest distance’ was defined by the nearest distance of the nucleus of cell type B (nearest cell, NC) to the nucleus of cell type A (reference cell, RC) (Figure 2a). The nearest distances for two representative patient samples (Patient 2433 and Patient 7422) are shown in Figure 2b. Coloured lines represent the percentages of the RC that was within the nearest distance, ranging from 10 to 150 μm, to each NC (Figure 2b). These data indicate that the spatial relationship between cells was heterogeneous within each sample from the same patient as well as between different patients’ samples. The ‘median intercellular nearest' (MIN) distance was further used as a parameter to represent the heterogeneity of cell–cell spatial distributions in each sample. The MIN distance was defined as the median value of the nearest distance for the RC‐to‐NC across high magnification images acquired in the same tissue specimen. For instance, when CD4^+^FOXP3^+^ T cells were used as the RC, the MIN distance of tumor cells was 11.12 μm of P2433 and 28.83 μm of P7422; the MIN distance, of CD8^+^ T cells, was 19.60 μm of P2433 and 8.6 μm of P7422 (Figure 2b). These data indicate that CD4^+^FOXP3^+^ T cells were closer to tumor cells in P2433, but closer to CD8^+^ T cells in P7422. Representative nearest distance analyses where the RC were CD8^+^ T cells, as well as tumor cells, are shown in Figure 2b. Collectively, this MIN distance analysis would likely have functional implications as a distance < 20 μm between cells represents direct cell–cell contact based on the average size of tumor cells of 20–30 μm and an average size of lymphocytes of 10 μm.^17^

T‐cell distribution within colorectal cancer was previously described using the regions ‘tumor core’ and ‘tumor margin’.^6^, ^18^ We assessed the densities of different T‐cell subsets, CD56^+^ cells and lineage^−^ cells in both the tumor core (average 20 images for each sample) and tumor edge (average 6 images for each sample). Within the 48 samples, 21 had tumor edge images available for further analysis. We observed the densities of CD8^+^ T cells and CD4^+^ T cells in the tumor core were significantly less compared to the tumor edge (Supplementary figure 1a, left panel). There was no statistical difference in the densities of CD4^+^FOXP3^+^ T cells, DNT cells, CD56^+^ cells and lineage^−^ cells between the tumor core and edge (Supplementary figure 1a, left panel). In addition, CD8^+^ T cells, CD4^+^FOXP3^+^ T cells and DNT cells showed a significant difference in the immune cell: tumor cell ratio between the tumor core and edge (Supplementary figure 1a, right panel). Collectively, the density of T cells, especially CD8^+^ T cells, showed a significant difference between the tumor core and edge. ISAT was then used to calculate the immune cell to tumor cell MIN distance; however, there was no difference in this parameter between the tumor core and tumor edge (Supplementary figure 1b). This may suggest that immune cells infiltrating the tumor core represent an effective immune response from the host.

### Increased numbers of T‐cell subsets in the GC tumor core correlated with better patient survival

**Figure 3:**
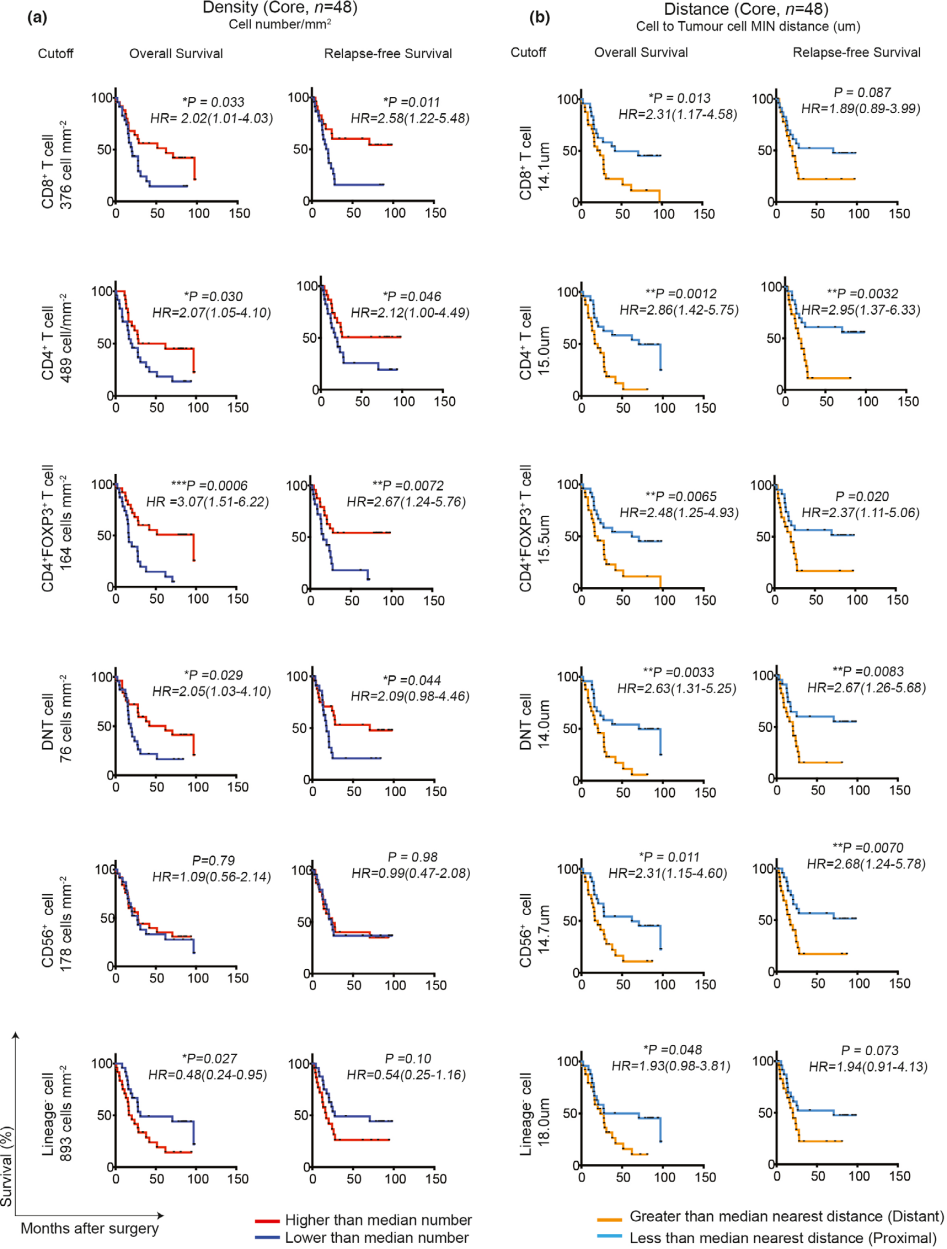
Higher densities of T‐cell subsets, especially CD4^+^FOXP3^+^ T cells, in the tumor core correlate with better patient survival. **(a)** Overall survival (OS; left) and relapse‐free survival (RFS; right) of all 48 patients based on densities of CD8^+^ T cells, CD4^+^ T cells, CD4^+^FOXP3^+^ T cells, DN T cells, CD56^+^ cells and Lineage^−^ cells. Individual immune infiltrate values were divided into higher (*n* = 24) or lower (*n* = 24) based on the median number of cells. **(b)** OS (left) and RFS (right) analysis of all 48 patients based on MIN distance to tumor cells from CD8^+^ T cells, CD4^+^ T cells, CD4^+^FOXP3^+^ T cells, DN T cells, CD56^+^ cells and Lineage^−^ cells. Distant (*n* = 24) and proximal (*n* = 24) distance values were defined based on the MIN distance. Hazard ratio (HR) is shown for lower compared to higher for density analysis, and distant compared to proximal for distance analysis. HR and 95% confidence interval are shown. Significance was determined using the log‐rank Mantel–Cox test. *P < 0.05, **P < 0.01. DNT cell: Double‐negative T cell.

To define whether the numbers of these tumor‐infiltrating immune subsets correlate with patient survival, overall survival (OS) was defined as the time period from curative surgery to death, without specified cause of death. Relapse‐free survival (RFS) was defined as the time period from curative surgery to clinically detectable recurrence. Patients were stratified into low‐ or high‐level groups based on the median number of the immune cells. We observed high levels of tumor‐infiltrating T‐cell subsets, including CD8^+^ T cells, CD4^+^ T cells, CD4^+^FOXP3^+^ T cells and DNT cells, were associated with prolonged OS and RFS (Figure 3a). In contrast, infiltration of lineage^−^ cells was associated with poorer OS but not with RFS (Figure 3a). Collectively, these data highlight the clinical relevance of tumor‐infiltrating T cells for patients’ survival. The densities of each immune subset from the tumor edge were also analysed, where tumor edge images were available (*n* = 21). The data showed a similar trend to the tumor core, but no significant association with OS and RFS was observed (Supplementary figure 2a), which is likely to be due to the limited number of samples. In addition, tumor edge is more difficult to interpret anatomically especially for diffuse gastric cancer and hence would provide less consistent information.

T cells require proximity to their target cells in order to carry out cytotoxic functions, and T cells in proximity to each other form effective signalling networks via cytokine secretion. To explore whether spatial relationships between immune cells and tumor cells had prognostic significance, patients were stratified into proximal and distant groups based on the MIN distance between each cell subpopulation and the tumor cells. We observed that close interactions between either T‐cell subpopulations or CD56^+^ cells and tumor cells were associated with improved OS and RFS (Figure 3b). These data indicate that immune cell–tumor cell interactions have functional relevance or represent a strong host immune response. The MIN distance of each immune subset to tumor cells was also analysed in 21 samples where tumor edge images were available. There was a similar trend as observed from the tumor core, but no significant correlation with either OS or RFS for MIN distances at the tumor edge was observed (Supplementary figure 2b), due to a limited number of the tumor edge cohort.

### CD8^+^ T‐cell number and proximity correlated with a high number of tumor‐infiltrating CD4^+^FOXP3^+^ T cells in GC

To determine independent prognostic factors that may be useful in predicting the survival outcome of GC patients, we used all the immunological variables from Figure 3 that reached significance in univariate analysis, in combination with established clinical variables to create a multivariate Cox regression model for OS. In multivariate analysis, we found that a higher number of CD4^+^FOXP3^+^ T cells in the tumor core was an independent predictor of prolonged survival (HR = 0.238, *P* < 0.001, Supplementary table 1). The association of good prognosis with CD4^+^FOXP3^+^ T cells was unexpected and counter‐intuitive as this phenotype is generally considered to be immune‐suppressive CD4^+^ regulatory T cells (Treg).^19^, ^20^ However, in some cancers, including colorectal cancer,^21^ a high number of tumor‐infiltrating CD4^+^FOXP3^+^ T cells was shown to be associated with improved survival.^22^ One possible explanation was that CD4^+^FOXP3^+^ T cells in humans are functionally and phenotypically heterogeneous, including both suppressive and non‐suppressive functions.^22^, ^23^

**Figure 4:**
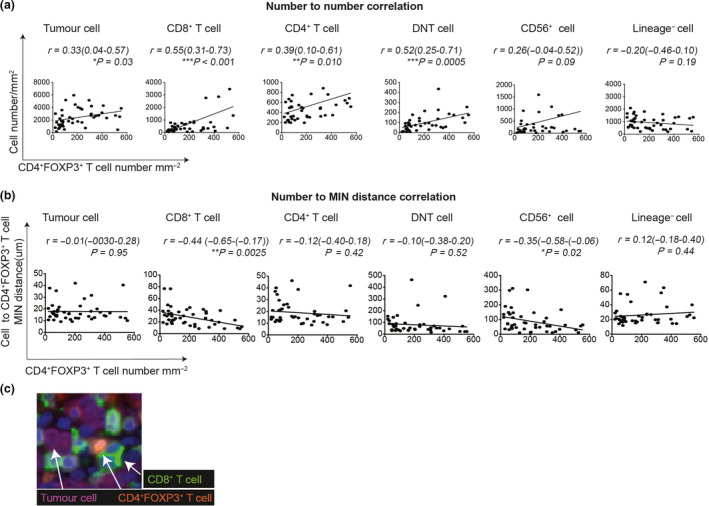
Close interactions between CD4^+^FOXP3^+^ T cells and CD8^+^ T cells, but not tumor cells. **(a)** Correlation analysis (*n* = 48) between CD4^+^FOXP3^+^ T cells density and densities of tumor cells, CD8^+^ T cells, CD4^+^ T cells, DNT cells, CD56^+^ cells and Lineage^−^ cells. **(b)** Correlation analysis (*n* = 48) between the CD4^+^FOXP3^+^ T cells density and the MIN distance from CD4^+^FOXP3^+^ T cells (as the RC) to tumor cells, CD8^+^ T cells, CD4^+^ T cells, CD56^+^ cells, DNT cells and Lineage^−^ cells. Pearson correlation coefficient (*r*), 95% confidence interval and significance levels (*P*‐value) are shown. **(c)** Example image showing close interactions between CD4^+^FOXP3^+^ T cells (orange) and CD8^+^ T cells (green), not tumor cells (magenta). *P < 0.05, **P < 0.01, ***P < 0.001. DNT cell: Double‐negative T cell.

To examine which immune subset(s) was interacting with CD4^+^FOXP3^+^ T cells in GC tumors, we performed correlation analysis between densities of other immune cells subsets and CD4^+^FOXP3^+^ T cells. We observed a positive and consistent correlation between the T‐cell subpopulations (CD8^+^, CD4^+^ and DNT cells) and CD4^+^FOXP3^+^ T cells (Figure 4a). We then explored whether these T‐cell subsets were in direct cell–cell contact with CD4^+^FOXP3^+^ T cells using the ISAT tool. In this analysis, CD4^+^FOXP3^+^ T cells were used as the RC and other cell subsets as the NC. If the NC population is randomly distributed relative to the RC, the MIN distance will not correlate with the number of RC. We observed an increasing number of CD4^+^FOXP3^+^ T cells in the tumor do not correlate with a reduced MIN distance between CD4^+^FOXP3^+^ T cells and tumor cells (*r* = −0.01, *P* = 0.95, Figure 4b). This result indicates that CD4^+^FOXP3^+^ T cells were randomly distributed in relation to tumor cells, despite the density of CD4^+^FOXP3^+^ T cells. Similar results were observed between CD4^+^FOXP3^+^ T cells and CD4^+^ T cells, as well as between CD4^+^FOXP3^+^ T cells and DNT cells (Figure 4b). However, the MIN distance between CD8^+^ T cells and CD4^+^FOXP3^+^ T cells, with the median cut‐off of 23.65 μm, was significantly reduced with an increased number of CD4^+^FOXP3^+^ T cells (*r* = −0.44, *P* = 0.0025, Figure 4b). This reflects an increasing number of CD4^+^FOXP3^+^ T cells correlates with close proximity with CD8^+^ T cells. In addition, the MIN distance between CD56^+^ cells and CD4^+^FOXP3^+^ T cells was observed to be correlated with CD4^+^FOXP3^+^ T‐cell numbers (*r* = −0.35, *P* = 0.02, Figure 4b). However, the median MIN distance was 63.5 μm, indicating that this interaction is more likely to represent an indirect mechanism of interaction. Collectively, these data show that an increased number of CD4^+^FOXP3^+^ T cells was associated with an increased number and proximity to CD8^+^ T cells within the tumor core (example image is shown in Figure 4c). This finding is aligned with previous reports using a double‐staining approach of CD8^+^ and FOXP3^+^ T cells in GC^24^ and rectal cancer.^25^ CD4^+^FOXP3^+^ T cells, in combination with CD8^+^ T cells, may perform an important biological function leading to better patient outcomes.

### A combined analysis of intra‐tumoral CD8^+^ T cells and CD4^+^FOXP3^+^ T cells is an independent biomarker of good prognosis

**Figure 5:**
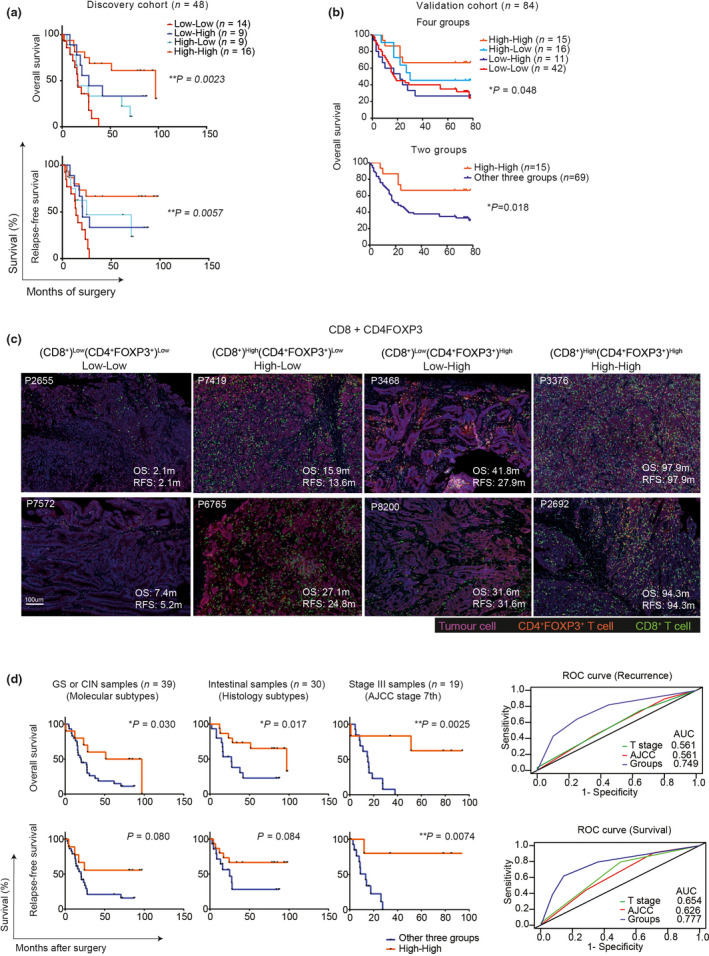
CD8 + CD4FOXP3 grouping as an independent biomarker of patients’ prognosis. **(a)** OS and RFS in the discovery cohort (*n* = 48) showing differential co‐existence of CD8^+^ T cells and CD4^+^FOXP3^+^ T cells, stratified into (CD8^+^)^Low^(CD4^+^FOXP3^+^)^Low^ (the Low‐Low group), (CD8^+^)^High^(CD4^+^FOXP3^+^)^Low^ (the High‐Low group), (CD8^+^)^Low^(CD4^+^FOXP3^+^)^High^ (the Low‐High group) and (CD8^+^)^High^(CD4^+^FOXP3^+^)^High^ (the High‐High group). Significance was determined using the log‐rank Mantel–Cox test. **(b)** OS of patients in an independent international validation cohort (*n* = 84) showing differential co‐existence of CD8^+^ T cells and CD4^+^FOXP3^+^ T cells, stratified into four and two groups, respectively. (**c**) Representative images of GC tumor showing differential co‐localisation of CD8^+^ (green) and CD4^+^FOXP3^+^ T cells (orange) were associated with short, median, and prolonged OS and RFS, respectively. **(d)** Subgroup analysis to estimate the OS and RFS of patients according to molecular subtypes (*n* = 39), histology subtypes (*n* = 30) and AJCC stage (*n* = 19), respectively. Significance was determined using the log‐rank Mantel–Cox test. **(e)** ROC curve using CD8 + CD4FOXP3 groups (Groups), T stages and AJCC stages to predict recurrence or survival. *P < 0.05, **P < 0.01, ***P < 0.001. DNT cell: Double‐negative T cell.

Further exploration of the interaction between CD4^+^FOXP3^+^ T cells and CD8^+^ T cells may improve our understanding of tumor‐specific T‐cell activity in the tumor microenvironment, with potential impact on the development of immune‐modulatory therapies. To explore this relationship further, a combined analysis of CD8^+^ T cells and CD4^+^FOXP3^+^ T cells (referred to as CD8 + CD4FOXP3) was performed. Based on the median cell densities, GC tumors were stratified into (CD8^+^)^Low^(CD4^+^FOXP3^+^)^Low^ (defined as Low‐Low), (CD8^+^)^High^(CD4^+^FOXP3^+^)^Low^ (defined as High‐Low), (CD8^+^)^Low^(CD4^+^FOXP3^+^)^High^ (defined as Low‐High) and (CD8^+^)^High^(CD4^+^FOXP3^+^)^High^ (defined as High‐High) groups. The median threshold for CD8^+^ T cells was 376 cells per mm^2^, and for CD4^+^FOXP3^+^ T cells was 164 cells per mm^2^, in line with the cut‐offs in Figure 3a. Patients in the High‐High group had significantly prolonged OS with a median OS of 97.03 months when compared to patients in the other three groups (median OS: 27.77 months in the Low‐High group, 15.90 months in the High‐Low group and 16.32 months in the Low‐Low group) (Figure 5a and Supplementary table 2). A similar finding was observed for RFS with the median RFS unreached in the High‐High group, 20.6 months in the Low‐High group, 24.87 months in the High‐Low group, and 14.03 months in the Low‐Low group (Figure 5a and Supplementary table 2). We further validated this result in an independent international cohort using a tissue microarray (TMA) comprising 84 gastric tumor core samples by mIHC of CD8^+^, FOXP3^+^ and PD‐L1^+^ cells. We applied the same thresholds as the discovery cohort (376 cells per mm^2^ for CD8^+^ T cells and 164 cells per mm^2^ for FOXP3^+^ T cells) in the validation cohort. Similarly, we observed a significantly prolonged OS in the High‐High group when compared to the other three groups (Figure 5b). Collectively, these results indicate CD8 + CD4FOXP3 grouping is a valuable prognostic marker for GC patients’ stratification. Representative images of the densities of CD8^+^ T cells and CD4^+^FOXP3^+^ T cells, as well as patients’ OS and RFS in the four groups, are shown in Figure 5c.

To further evaluate the independent predictive ability of CD8 + CD4FOXP3 in predicting GC patients’ survival, we combined the CD8 + CD4FOXP3 with other GC clinicopathologic factors as categorical variables in a Cox proportional hazards model. The clinicopathologic factors included American Joint Committee on Cancer (AJCC) staging, Lauren classification and molecular subtypes. We observed that only AJCC and CD8 + CD4FOXP3 remained significant for OS and RFS in the multivariate analysis (Supplementary table 3). Furthermore, we performed subgroup analysis in patients with the same clinicopathologic factors, such as the same molecular subtypes (CIN or GS samples, *n* = 39), the same histological subtype (intestinal subtype, *n* = 30) or the same AJCC stage patients (stage III, *n* = 19). We observed the CD8 + CD4FOXP3 grouping discriminated the OS and RFS between the High‐High group and the other three groups (Figure 5d). To measure the performance of this CD8 + CD4FOXP3 model as a predictor of patient outcome, we performed receiver operating characteristic (ROC) curve analysis in the discovery cohort. The CD8 + CD4FOXP3 groups had higher AUC values than T‐stage or AJCC staging for GC patient recurrence and survival (Figure 5e). These data indicate CD8 + CD4FOXP3 grouping is a significant prognostic biomarker for GC patients.

### An up‐regulation of PDL1 and interferon‐gamma (IFN‐γ) response was found in the High‐High tumors

**Figure 6:**
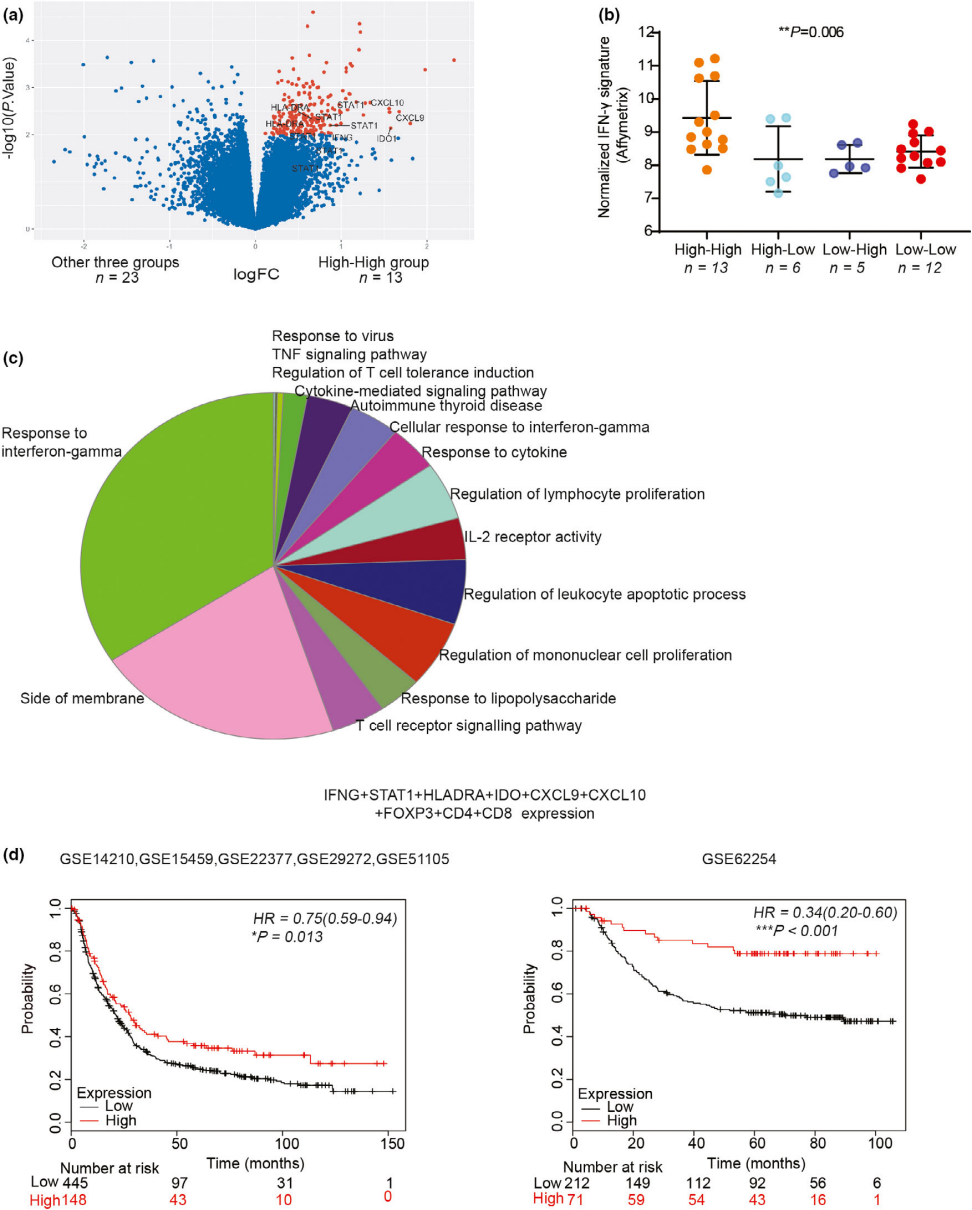
Association of interferon‐gamma (IFN‐γ) response and the High‐High GC tumors. **(a)** A volcano plot depicts the differentially expressed genes in the High‐High group (*n* = 13) when compared to the other three (CD8 + CD4FOXP3) groups (*n* = 23). Genes up‐regulated in the High‐High group and with *P*‐value < 0.01 are labelled in red. Genes associated with IFN‐γ response (CXCL9, CXCL10, IDO1, IFNG, HLA‐DRA and STAT1) are labelled. **(b)** IFN‐γ signature expression in four groups (*n* = 13, 6, 5, 12). **(c)** Nodes are representing enriched pathways with *P*‐value < 0.01 based on shared genes in pie chart format. **(d)** The kmplot interface 26 with overall survival data in two separate cohorts (*n* = 593 and *n* = 283) of GC patients was interrogated. Patients were classified into high (upper quartile) and low expressing groups based on the mean expression of the selected genes. HR is shown for high compared to the low mean expression of genes. *P < 0.05, **P < 0. 01, ***P < 0.001.

To explore whether this unique immune cell clustering in the High‐High tumors was associated with changes in the tumor immune signalling networks, we analysed the gene expression profile of 36/48 GC patient samples, where data were available. These 36 GC patient samples were classified into four sub‐groups, as described in Figure 5a. A volcano plot (Figure 6a) showed the differential gene expression between the High‐High group and the other three groups. Of note, the IFN‐γ‐related gene signature (CXCL9, CXCL10, IDO1, IFNG, HLA‐DRA and STAT1) was significantly increased in the High‐High group (labelled in Figure 6b & Supplementary figure 3). To further analyse the putative function of genes up‐regulated in this group, gene ontology analysis was performed using the significantly up‐regulated probe sets in Figure 6a (*n* = 186, *P* < 0.01). The High‐High tumors were significantly increased with genes active in immune signalling pathways (pathways with *P* < 0.01, Figure 6c and Supplementary figure 4), with the response to IFN‐γ pathway highlighted. This suggests the High‐High group was associated with GC tumors with an active adaptive immune response. While no significant difference in T‐stage or invasive potential was found, we did observe less nodal metastases in this High‐High group (Supplementary figure 5a and b), which warrants further investigation.

Finally, we validated the predictive power of the High‐High GC gene signature using a GC mRNA public database.^26^ We observed the gene signature (CXCL9 + CXCL10 + IDO1 + IFNG + HLADRA + STAT1 + FOXP3 + CD4 + CD8) predicted prolonged survival of patients in larger GC cohorts (Figure 6d). The gene signature is consistent with the signature previously described in the responders to ICB therapy targeting the PD1/PDL1 pathway.^5^, ^27^ In light of the presence of IFN‐γ response in the High‐High tumors, we further investigated the expression of PDL1 in these tumors. PDL1 expression in the tumor tissue was up‐regulated in the High‐High tumors when compared to the Low‐Low tumors by transcriptome analysis (Supplementary figure 6a), and mIHC in the discovery cohort (Supplementary figure 6b and c) and the validation cohort (Supplementary figure 6d). These results suggest that GC patients with both high levels of CD8^+^ T cells and CD4^+^FOXP3^+^ T cells may benefit from anti‐PD1/PDL1 therapy.

### The High‐High group has evidence of immune activation in peripheral blood compared to the Low‐Low group

**Figure 7:**
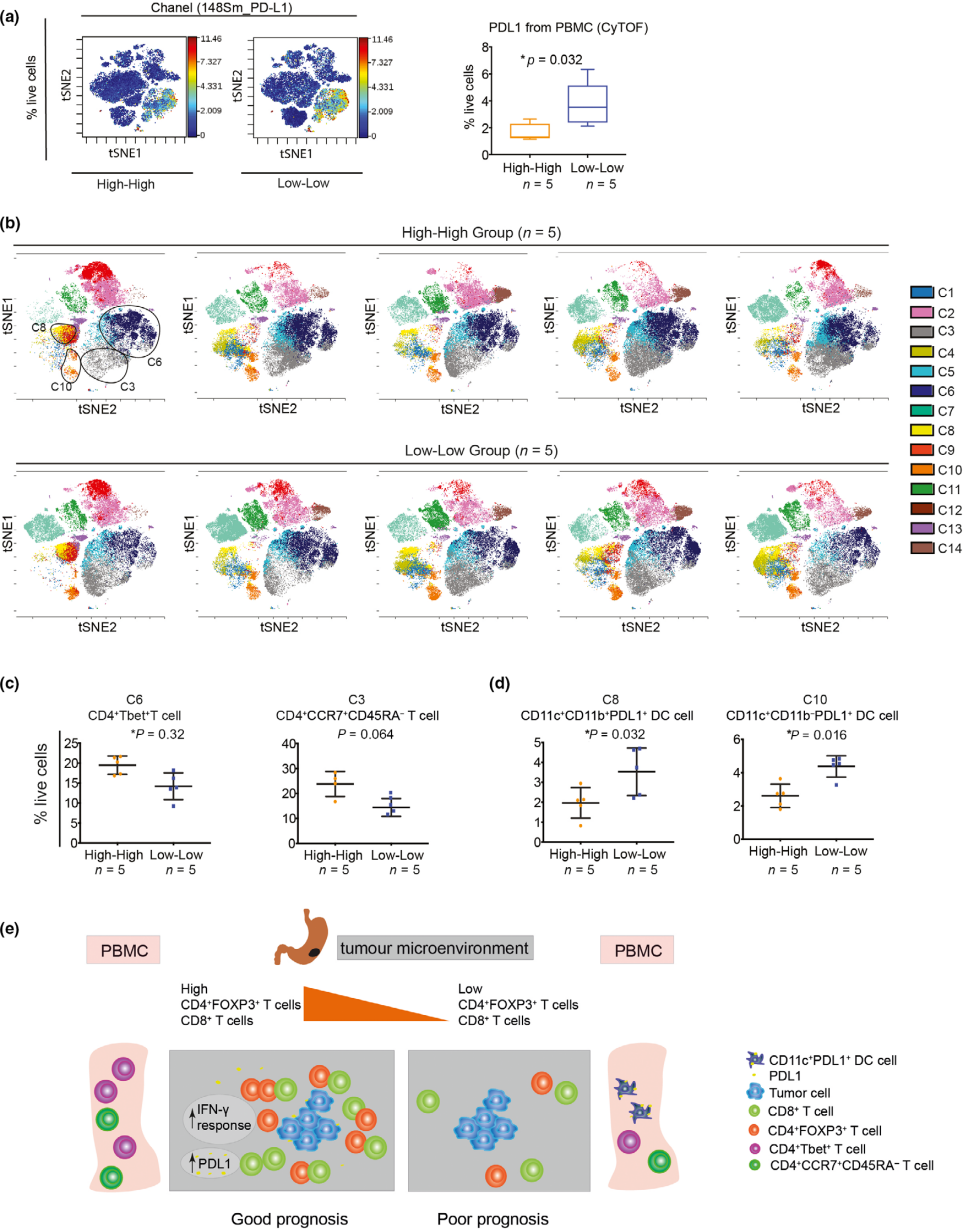
The High‐High GS/CIN tumors were associated with significantly increased Tbet^+^ CD4^+^ T cells and central memory CD4^+^ T cells in the peripheral blood. **(a)** PDL1 expression by cells in the peripheral blood was up‐regulated in the Low‐Low group using mass cytometry. Significance was determined using the two‐tailed Mann–Whitney *U*‐test. **(b)** viSNE illustration of 14 clusters identified by SPADE in the High‐High group (*n* = 5) and the Low‐Low group (*n* = 5). **(c, d)** Within the 14 clusters, Tbet^+^ CD4^+^ T cells (C6) and CCR7^+^CD45RA^−^ CD4^+^ T cells (C3) were significantly increased in the High‐High group **(c)**, while two CD11c^+^ dendritic cells clusters were significantly increased in Low‐Low group **(d)**. Significance was determined using the two‐tailed Mann–Whitney *U*‐test. Data are presented as mean ± SD. *P < 0.05, **P < 0.01, ***P < 0.001. **(e)** Our working model depicts how immune tumor microenvironment and peripheral blood in the GC can be interpreted to enable prognostication of patients.

As shown in Figure 5d, GS and CIN (GS/CIN) patients with the High‐High phenotype also had excellent survival. GS/CIN tumors represent the majority of GC patients (70% combined)^2^; no prior data have shown these two groups were associated with activated host immunity and could benefit from the current immunotherapy strategies. To determine whether GS/CIN patients with the High‐High phenotype had co‐existing systemic immunity which leads to improved immune‐surveillance, we analysed the peripheral blood from five High‐High patients and five Low‐Low patients using mass cytometry (CyTOF). High‐dimensional analysis with the viSNE algorithm showed an increased PDL1 expression in peripheral blood of the Low‐Low group (Figure 7a). The viSNE and SPADE algorithms were used to subdivide the peripheral blood immune cells into 14 distinct clusters (Figure 7b & Supplementary figure [Link], [Link]). Within the 14 clusters, effector CD4^+^ T cells (C6) and circulating central memory CD4^+^ T cells (C3) were increased in the peripheral blood of the High‐High group when compared to the Low‐Low group (Figure 7c). This suggests a robust CD4^+^ T‐cell peripheral blood compartment was present in patients with good outcome and is consistent with an anti‐tumor immune response as described elsewhere ^28^, ^29^. In contrast, the Low‐Low group patients’ peripheral blood had more CD11c^+^ dendritic cells (clusters C8 and C10, Figure 7d). Notably, there were also more PDL1^+^ cells contained within clusters C8 and C10 (Supplementary figure [Link], [Link]), suggesting a tolerogenic‐like cell‐mediated immune response^30^ in the peripheral blood of the Low‐Low patients. Other clusters, including B cells (C7), CD56^+^ cells (C11), CD8^+^ T cells (C2 and C9), CD4^+^CCR7^−^CD45RA^−^ cells (C5) and monocytes (C1, C4 and C12), did not show significant differences between the two groups (Supplementary figure 8a).

We compared differentially expressed genes between the High‐High GS/CIN tumors with other GS/CIN tumors and performed GO analysis to reveal active immune signalling pathways. We found the High‐High GS/CIN tumors were significantly increased with genes active in antigen processing and presentation, IFN‐γ response and DC differentiation (Supplementary figure 8b), suggesting a pre‐existing spontaneous tumor‐specific immune response in the local tumors.

In conclusion, our study showed that increased numbers of CD4^+^FOXP3^+^ T cells and CD8^+^ T cells (High‐High) in GC patients were associated with a good prognosis. These patients had an increased IFN‐γ response gene signature and PD‐L1 up‐regulation, plus increased frequencies of effector and central memory CD4^+^ T cells in the peripheral blood (Figure 7e). In contrast, GC tumors with low CD4^+^FOXP3^+^ T‐cell and CD8^+^ T‐cell density (Low‐Low) have a significantly increased number of CD11c^+^ dendritic cells in the peripheral blood.

## Discussion

In this study, we investigated the immune cells in the GC tumor microenvironment and explored their associations with patient outcomes. We investigated the GC immune context by combining immune cellular characteristics and gene expression profile data. We derived high‐dimensional GC immune cellular characteristics by combining immune cells densities as well as intercellular spatial relationships using our novel ISAT algorithm. This study revealed an independent prognostic biomarker, CD8 + CD4FOXP3, for GC patients. The association of CD4^+^FOXP3^+^ T cells with prolonged survival was unexpected and seemed counter‐intuitive as this phenotype has traditionally been associated with immune‐suppressive CD4^+^ regulatory T cells (Tregs). Indeed, the CD4^+^FOXP3^+^ T cells include both natural Treg cells (nTreg)^31^ and peripheral‐induced Treg (iTreg)^32^ cells. It is recognised that FOXP3 expression is required for their immune‐suppressive function.^33^ Numerous correlative studies have revealed that the density of tumor‐infiltrating Treg cells has prognostic significance for some cancers,^34^ suggesting that Treg cells may have a functional impact on tumor development and progression. The finding that a high density of infiltrating FOXP3^+^ Treg cells was associated with unfavorable outcome in many cancers supported the theory that tumor‐infiltrating FOXP3^+^ Treg cells were suppressing the anti‐tumor response and enabling cancer immune escape. The association was particularly strong for ovarian cancer^35^ and renal cancer.^36^ In contrast, in some cancers, especially follicular lymphoma^37^ and gastrointestinal cancers,^38^ such as colorectal cancer,^21^ a high number of tumor‐infiltrating FOXP3^+^ Treg cells was associated with improved overall survival. An association between a high number of tumor‐associated FOXP3^+^ T cells and improved survival was also observed in this study.

One possible explanation for our observation is the increasing evidence that CD4^+^FOXP3^+^ T cells in humans are functionally and phenotypically heterogeneous, including both suppressive and non‐suppressive function.^22^, ^23^ In certain cancers, CD4^+^FOXP3^+^ T cells in tumor tissue were classified into two functional subtypes by the level of FOXP3 expression.^22^ A high number of ‘low FOXP3 expressing’ CD4^+^ T cells (non‐Treg) correlated with a significantly better prognosis than those with predominantly ‘high FOXP3 expression’ CD4^+^ T cells infiltration. Such CD4^+^ T cells with low expression of FOXP3 produced significant amounts of IFN‐γ after *in vitro* stimulation.^22^ We observed these non‐Treg CD4^+^FOXP3^+^ T cells exist in the gastric tumor tissue (data not shown) and up‐regulation of IFN‐γ response genes in the High‐High tumors. This may explain why, in our study, samples with a high CD4^+^FOXP3^+^ T cells number showed a superior prognosis. As it is not possible to distinguish between the CD4^+^ T cells with low and high expression of FOXP3 in tumor tissues by conventional immunohistochemistry, this remains a limitation of our study. However, it may have been a major confounding factor in previous studies showing conflicting correlations with prognosis for CD4^+^FOXP3^+^ T cells in cancer.

To exert such a powerful prognostic influence on patient outcome, we explored the hypothesis that intra‐tumoral CD4^+^FOXP3^+^ T cells in GC interact with other nearby immune effector cells and exert their anti‐tumor effect indirectly rather than via direct tumor cell contact. Using the novel ISAT algorithm, we observed that a high number of CD4^+^FOXP3^+^ T cells interacting closely with CD8^+^ T cells, but not the tumor cells. This closed interaction in the tumor microenvironment was associated with better OS and RFS of GC patients. The MIN distance (< 20 μm) between CD4^+^FOXP3^+^ T cells and CD8^+^ T cells indicates direct cell‐to‐cell contact. This finding is in keeping with previous reports using a double‐staining approach of CD8^+^ and FOXP3^+^ T cells in GC^24^ and rectal cancer.^25^ In our study, we used the multi‐parameter nature of multiplex IHC and imaging, plus the novel ISAT algorithm, and revealed a close spatial relationship between CD4^+^FOXP3^+^ T cells and CD8^+^ T cells, but not other immune subsets.

Furthermore, we discovered the GC microenvironment with increased CD4^+^FOXP3^+^ T cells and CD8^+^ T cells (the High‐High group) had a robust IFN‐γ response and PDL1 expression, suggesting a strong immune activation in the tumor. Recent studies observed that IFN‐γ response could be a biomarker^5^, ^39^, ^40^ for ICB. Our results provide evidence that patients with increased CD4^+^FOXP3^+^ T cells and CD8^+^ T cells will not only have a favorable prognosis but may also be candidates for ICB as a result of high IFN‐γ and PDL1 expression in the tumor. We observed the High‐High group was associated with PDL1 up‐regulation in the tumor, Tbet^+^ CD4^+^ T cells circulating in the peripheral blood and a gene signature characterised by a robust IFN‐γ response, antigen processing and presentation via MHC‐I pathways. This group of patients had better overall survival and a significantly lower recurrence rate (RFS). One explanation for this observation is that this group of patients may develop a local tumor immunity that generates a systemic anti‐tumor immune response, which may reduce metastatic burden by an active circulating anti‐tumor immunity. In support of this hypothesis, we observed increased effector CD4^+^ T cells (Tbet^+^ CD4^+^ T cells) and reduced tolerogenic DCs in the peripheral blood of the high‐high group.

In conclusion, our study showed an increased number of CD4^+^FOXP3^+^ T cells, which clustered with CD8^+^ T cells in GC patients, was associated with a good prognosis. This finding contributes to the growing knowledge of non‐Treg CD4^+^FOXP3^+^ T‐cell function in cancer. Direct contact between CD4^+^FOXP3^+^ T cells and CD8^+^ T cells revealed a synergistic effect with a robust IFN‐γ response and PDL1 overexpression in GC. CD8 + CD4FOXP3 grouping provides an independent prognostic indicator, which can be used to stratify GC patients with a good outcome and possibly responsive to ICB, irrespective of microsatellite instability (MSI) or Epstein–Barr virus (EBV) status. We further observed patients with increased numbers of CD4^+^FOXP3^+^ T cells and CD8^+^ T cells in the tumor core have strong systemic immunity, which may be the mechanism of improved survival. In contrast, patients without this enrichment have a more immunosuppressive peripheral blood milieu that may be more permissive to metastases. This remains to be confirmed in prospective studies but may lead to non‐invasive biomarkers that could predict response to ICB and improve our chances of creating a functional bioassay for precision immunotherapy.

## Methods

### Patient cohorts

This study was approved by the Institutional Ethics Committee at the Peter MacCallum Cancer Centre, Melbourne, Australia (PMCC HREC 12/15). Patients were enrolled between 1999 and 2009 in the study from 10 hospitals in the Melbourne metropolitan area of Australia. Forty‐eight GC patients from the cohort^41^ who met the inclusion criteria (no neoadjuvant therapies before curative gastrectomy) were analysed. All 48 GC tissues were formalin‐fixed and paraffin‐embedded. Informed consent was obtained from all patients. Clinical information was recorded at enrolment. All available medical records and patient questionnaires for the cohort were reviewed. Outcome data for the clinical database of this cohort were last updated in December 2012. Pathological review of tumor tissue has been performed to confirm the presence of a tumor and histological subtype of the tumor.

The validation cohort was obtained from Shanghai Jiao Tong University, Ruijin Hospital. GC tissues were formalin‐fixed and paraffin‐embedded. All protocols using human specimens were approved by Shanghai Jiao Tong University Human Ethics Committee, and informed consent was obtained from all patients. One tissue microarray (TMA) included 90 GC tumor core tissues with each core diameter 1.5 mm from 90 patients^42^ (6 samples were excluded because of the incompleteness of the tissues).

### Seven‐colour multiplex immunohistochemistry (mIHC)

Multiplex IHC staining was performed using the Opal 7‐colour kit (PerkinElmer, Waltham, MA, USA) per manufacturer's instructions. Four μm sections from FFPE tissue blocks were de‐paraffinised and rehydrated before antigen retrieval (EDTA pH 8.0) with a pressure cooker. Tissue sections were blocked with serum‐free protein block (Dako, Glostrup, Denmark) for 10 min before applying each primary antibody (Supplementary table 4) for 30 min at room temperature. Endogenous peroxidase activity was blocked with H_2_O_2_ for 10 min (performed once, only after the first primary antibody). The HRP‐labelled anti‐IgG secondary antibody (PerkinElmer) was added at room temperature for 10 min. For visualisation, Opal TSA Plus (1:50) dye was applied on to the tissues for 10 min. Slides were placed in EDTA pH 8.0 buffer, and heat‐induced antigen retrieval (HIER) step performed using the microwave treatment. For each cell marker, repeat rounds of the steps above were repeated in sequence followed by a HIER step. For each round of mIHC staining, three washes (1× TBST, 0.5% Tween‐20) were performed between each step, except after the Opal TSA Plus dye, where five washes were performed. After the final antigen retrieval using the microwave, the slides were washed twice before the nuclei were stained with 4′,6‐diamidino‐2‐phenylindole solution (1:250; PerkinElmer) and coverslipped using the Vectashield HardSet mounting medium (Vector Labs, Burlingame, CA, USA).

For the validation cohort mIHC staining, Opal 4‐colour fluorescent IHC kit (PerkinElmer) with PD‐L1, FOXP3 and CD8 multiplex IHC antibody panel (#78701 kit; Cell Signaling Technology, Danvers, MA, USA) was used. The TMA slide was scanned using a Nikon C1 confocal microscope (Nikon, Minato City, Tokyo, Japan). The positive stained CD8 and FOXP3 cell numbers per mm^2^, and the average intensity of PD‐L1 per mm^2^ was analysed by ImageJ software (ImageJ 1.51 n; National Institutes of Health, United States).^42^

### Multispectral imaging

Multiplex stained slides were imaged using the Vectra Multispectral Imaging System version 2 (PerkinElmer). Regions of interest were selected by 2 × 2 (1338 μm × 1000 μm) stamp and divided into tumor core and tumor edge. The tumor edge was defined as within 1mm interface between tumor and non‐malignant tissue. The tumor core area was defined as the proximal tumor area within the tumor edge. High magnification (20×) multispectral images were acquired to encompass all the regions for tumor core and tumor edge with minimum overlaps between images.

### Spectral unmixing and phenotyping

Using the multispectral images obtained from single stained slides for each marker, a spectral library containing fluorophores emitting spectral peaks was created (PerkinElmer). This spectral library was then used to separate each multispectral image into its components (spectral unmixing), which allows for the colour‐based identification of all seven markers in a single image using the inForm 2.2 image analysis software (PerkinElmer). All spectrally unmixed and segmented images were subsequently subjected to a proprietary inForm active learning phenotyping algorithm. This allows for the individual identification of each DAPI‐stained cell according to their pattern of fluorophore expression and nuclear/cell morphological features, associating their phenotype with specific x, y spatial coordinates. Cells were phenotyped into one of seven different subtypes according to our markers of interest as follows: tumor cells (AE1AE3^+^), CD8^+^ T cells (CD3^+^CD8^+^), CD4^+^ T cells (CD3^+^CD4^+^), CD4^+^FOXP3^+^ T cells (CD3^+^CD4^+^FOXP3^+^), double‐negative T cells (CD3^+^CD4^−^CD8^−^), CD56^+^ cells (CD56^+^) and Lineage^−^ cells (DAPI^+^). All phenotyping and subsequent quantifications were performed blinded to the sample identity and clinical outcomes.

### Density analysis and intercellular spatial analysis tool (ISAT)

An algorithm was developed in small batches using the PerkinElmer inForm and R software for cell phenotype density and distance analysis. A novel algorithm, termed the Intercellular Spatial Analysis Tool (ISAT), was jointly developed in 'R' by Minyu Wang and Yu‐Kuan Huang (https://cran.r‐project.org/web/packages/ISAT/index.html). ISAT was explicitly designed to calculate the spatial relationship between tumor cells and immune cells, or the spatial relationship between individual immune cell subsets within the GC tumor.^43^ The algorithm starts with a reference cell (RC, e.g. tumor) as the cell of origin, and calculates the distance to the nearest cell (NC) of a specific phenotype (e.g. CD8^+^ T cell). This information is collated for all spatial measurements between the designated RC and NC to derive the median intercellular nearest (MIN) distance. Public packages used in this algorithm include the following: 'gtools' and 'dplyr', 'rowr'.

### Tissue Affymetrix profiling

Ninety‐four GC samples were previously profiled using the Affymetrix U133 Plus 2 arrays and the data submitted to the Gene Expression Omnibus (GEO, Series GSE51105).^44^ Thirty‐six of the 48 tumor specimens characterised by mIHC had matched microarray data.

### Gene expression analysis

The differential gene expression between groups was analysed using the limma package.^45^ Genes were considered differentially expressed if *P*‐value ≤ 0.01. No correction was performed for multiple testing. Packages for graphics and data manipulation include the following: 'ggplot2', 'ggrepel', and 'dplyr'. Related pathways of the differential expressed genes were analysed with ClueGO^46^ and CluePedia^47^ plug‐ins of Cytoscape.^48^ All *P*‐values were two‐sided. A *P*‐value < 0.01 was considered statistically significant.

### PBMCs isolation and freezing

Human peripheral blood mononuclear cells (PBMCs) were isolated from 7 mL of venous blood, which was drawn before surgery, using the density gradient separation (Ficoll‐Paque™ Plus; GE Healthcare, Chicago, IL, USA). PBMCs were separated by low‐speed centrifugation of 400 *g* for 30 min, and this step was completed with the centrifuge ‘brake off’. PBMCs were then collected from the interphase layer and washed with RPMI 1640 medium (Gibco, Life Technologies, Carlsbad, CA, USA). Cell pellets were suspended in 1 mL freezing medium composed of 200 μL dimethyl sulfoxide (DMSO; Sigma‐Aldrich, St. Louis, MO, USA) and 800 μL foetal bovine serum (FBS, Gibco, Life Technologies) and transferred into 1.2 mL cryogenic vials (Sigma‐Aldrich), which were subsequently transferred into a freezing container (Thermo Fisher Scientific, Waltham, MA, USA) that had a cooling rate of −1°C per min. After 24 h at −80°C, cryogenic vials were transferred to liquid nitrogen (−196°C) for long‐term storage.

### PBMCs preparation for Cytometry by Time‐Of‐Flight (CyTOF)

Frozen PBMCs stored in cryogenic vials thawed in a 37°C water bath. After ice crystals have dissolved, the cell suspension was transferred into 15 mL tubes containing 10 mL of warm complete RPMI media, and the thawed cells were pelleted by centrifugation at 1500 rpm for 5 min at room temperature. The supernatant was removed, and the cell pellet was resuspended by tapping the tube. Cells were resuspended in 4 mL complete RPMI media with 80 μL of DNase (Thermo Fisher Scientific) and incubated in a 37°C 5% CO_2_ incubator for 15 min, after which the cells were topped up to 10 mL with RPMI media and centrifuged at 1500 rpm for 5 min at room temperature. The cell pellet was resuspended in complete RPMI media and rest for another 2 h at 37°C in a 5% CO_2_ incubator.

### CyTOF marker labelling and detection

One million viable rested cells from each patient were incubated with 50 μm cisplatin (Sigma‐Aldrich) for 3 min at room temperature. Cells were transferred into a FACS tube and washed once with Cell Staining Media (CSM; 2mm EDTA with 2% FBS, 0.05% Sodium Azide). Cells were then incubated for 30 min at 4°C with a 500 μL cocktail of metal conjugated surface antibodies. Cells were then washed, fixed and permeabilised according to the user's guide from Cell‐ID 20X‐Plex Pd Barcoding Kit (Fluidigm, South San Francisco, CA, USA). Each barcode was resuspended completely in 100 μL 1× Barcode Perm Buffer, transferred to the appropriated samples, and incubated for 30 min at room temperature. Barcoding aliquots were washed twice with CSM and pooled into one FACS tube. Cells were washed, fixed and permeabilised using the eBioscience Foxp3/Transcription Factor Fixation and Permeabilization Kit (Thermo Fisher Scientific) before incubated for 30 min at 4°C with a 500 μL cocktail of metal conjugated intercellular antibodies. The metal content of the antibodies used is listed in Supplementary table 5. Antibodies not purchased from fluidigm were purchased in purified form from the listed sources, and metal conjugated in house using the X8 Multi‐Metal Labelling Kit (Fluidigm). Total cells were identified by DNA intercalation (MaxPar^®^ Intercalator‐Ir; Fluidigm) in 2% PFA at 4°C overnight. Labelled cells were diluted in 1:10 dilution of 4 Element EQ calibration beads (Fluidigm) and assessed by the CyTOF Helios instrument (Fluidigm) using a flow rate of 0.030 mL min^−1^. All samples were processed and stained on the same day using barcode kits in the same experimental batch and then run on the Helios in the same batch. These samples were individually barcoded using the Cell‐ID 20X‐Plex Pd Barcoding Kit and then stained together in the sample tube to limit batch effects.

### Cell subset identification

Data files for each barcoded sample were concatenated using an in‐house script. The data were normalised using Normalizer v0.1 MCR.^49^ Files were de‐barcoded using the Debarcoder Software (Fluidigm). Debarcoded samples were analysed on the Cytobank platform^50^, ^51^ (https://www.cytobank.org/) by first performing gating of cell subsets followed by exclusion of debris (Iridium^−^; DNA^−^), cell doublets (Iridium^high^; DNA^high^) and dead cells (cisplatin^+^). Multidimensional data generated by CyTOF were assessed using viSNE and SPADE on the Cytobank platform. 38 017 cells from each sample with all markers in the CyTOF panel were used for SPADE clustering.

### Statistics

All statistical analyses were performed using the GraphPad Prism software (version 7.0d; GraphPad, San Diego, CA, USA) unless stated otherwise. Statistical analyses of quantifications were performed with the two‐tailed Mann–Whitney *U*‐test between two groups and the Kruskal–Wallis test among multiple groups as appropriate. Statistical analyses for PDL1 mIHC comparison were performed with a two‐sided chi‐square test. For survival analyses, Kaplan–Meier plots were drawn, and statistical differences were evaluated using the log‐rank Mantel–Cox test. Multivariate analyses of the survival data were performed for immune cell infiltration parameters and prognostic factors using a stepwise Cox regression analysis. The model was assessed for clinical relevance, and manual regression was performed by entering the most clinically relevant variable (from the list of variables in stepwise regression), and the change in hazard ratio, log‐likelihood and coefficient was observed before a clinically relevant model was developed. For the correlation analyses, Pearson's correlation coefficient (*r*) was calculated using the GraphPad Prism software. A *P*‐value of ≤ 0.05 was considered statistically significant.

## Conflict of interest

The authors declare no potential conflicts of interest.

## Author contributions

PJN and AB designed the project and contributed equally as senior authors supervising the project; ND collected samples; MW, HH, DT and HXA designed and optimised the experiments, and MW performed experiments; CM provided the pathology review; YL, FY and DX conducted experiments and analysis on the validation cohort. MW, YH and YS developed the analysis algorithm through R; MW, JK and IRG conducted the statistical analysis; and MW, LKM, PJN, RAB and AB wrote the paper. All authors discussed the results and their implications and commented on the manuscript at all stages.

## Supporting information

Supplementary figures 1‐5

Supplementary tables 1‐5

## Data Availability

The data that support the findings of this study are available from the authors on reasonable request, see author contributions for specific data sets. Code availability: The code of the ISAT algorithm can be accessed through https://cran.r‐project.org/web/packages/ISAT/index.html

## References

[1] Van Cutsem E , Sagaert X , Topal B , Haustermans K , Prenen H . Gastric cancer. Lancet 2016; 388: 2654–2664. 27156933 10.1016/S0140-6736(16)30354-3

[2] Cancer Genome Atlas Research N . Comprehensive molecular characterization of gastric adenocarcinoma. Nature 2014; 513: 202–209. 25079317 10.1038/nature13480 PMC4170219

[3] Waddell T , Verheij M , Allum W *et al* Gastric cancer: ESMO‐ESSO‐ESTRO Clinical Practice Guidelines for diagnosis, treatment and follow‐up. Ann Oncol 2013; 24(Suppl 6): vi57–vi63. 24078663 10.1093/annonc/mdt344

[4] Topalian SL , Hodi FS , Brahmer JR *et al* Safety, activity, and immune correlates of anti‐PD‐1 antibody in cancer. N Engl J Med 2012; 366: 2443–2454. 22658127 10.1056/NEJMoa1200690 PMC3544539

[5] Muro K , Chung HC , Shankaran V *et al* Pembrolizumab for patients with PD‐L1‐positive advanced gastric cancer (KEYNOTE‐012): a multicentre, open‐label, phase 1b trial. Lancet Oncol 2016; 17: 717–726. 27157491 10.1016/S1470-2045(16)00175-3

[6] Galon J , Costes A , Sanchez‐Cabo F *et al* Type, density, and location of immune cells within human colorectal tumors predict clinical outcome. Science 2006; 313: 1960–1964. 17008531 10.1126/science.1129139

[7] Galon J , Mlecnik B , Bindea G *et al* Towards the introduction of the 'Immunoscore' in the classification of malignant tumours. J Pathol 2014; 232: 199–209. 24122236 10.1002/path.4287 PMC4255306

[8] International Agency for Research on Cancer WG . International Agency for Research on Cancer: Lyon; 1994.

[9] Shibata D , Weiss LM . Epstein‐Barr virus‐associated gastric adenocarcinoma. Am J Pathol 1992; 140: 769–774. 1314023 PMC1886378

[10] Kim KJ , Lee KS , Cho HJ *et al* Prognostic implications of tumor‐infiltrating FoxP3^+^ regulatory T cells and CD8^+^ cytotoxic T cells in microsatellite‐unstable gastric cancers. Hum Pathol 2014; 45: 285–293. 24331841 10.1016/j.humpath.2013.09.004

[11] Lee JS , Won HS , Sun S , Hong JH , Ko YH . Prognostic role of tumor‐infiltrating lymphocytes in gastric cancer: a systematic review and meta‐analysis. Medicine (Baltimore) 2018; 97: e11769. 30095632 10.1097/MD.0000000000011769 PMC6133557

[12] Thompson ED , Zahurak M , Murphy A *et al* Patterns of PD‐L1 expression and CD8 T cell infiltration in gastric adenocarcinomas and associated immune stroma. Gut 2017; 66: 794–801. 26801886 10.1136/gutjnl-2015-310839 PMC4958028

[13] Haas M , Dimmler A , Hohenberger W , Grabenbauer GG , Niedobitek G , Distel LV . Stromal regulatory T‐cells are associated with a favourable prognosis in gastric cancer of the cardia. BMC Gastroenterol 2009; 9: 65. 19732435 10.1186/1471-230X-9-65 PMC2749861

[14] Zhou S , Shen Z , Wang Y *et al* CCR7 expression and intratumoral FOXP3+ regulatory T cells are correlated with overall survival and lymph node metastasis in gastric cancer. PLoS One 2013; 8: e74430. 24040244 10.1371/journal.pone.0074430 PMC3764061

[15] Perrone G , Ruffini PA , Catalano V *et al* Intratumoural FOXP3‐positive regulatory T cells are associated with adverse prognosis in radically resected gastric cancer. Eur J Cancer 2008; 44: 1875–1882. 18617393 10.1016/j.ejca.2008.05.017

[16] Postow MA , Manuel M , Wong P *et al* Peripheral T cell receptor diversity is associated with clinical outcomes following ipilimumab treatment in metastatic melanoma. J Immunother Cancer 2015; 3: 23. 26085931 10.1186/s40425-015-0070-4 PMC4469400

[17] Wood EJ . Cellular and molecular immunology (5th ed.): Abbas A. K., and Lichtman, A. H. Biochem Mol Biol Educ 2004; 32: 65–66.

[18] Anitei MG , Zeitoun G , Mlecnik B *et al* Prognostic and predictive values of the immunoscore in patients with rectal cancer. Clin Cancer Res 2014; 20: 1891–1899. 24691640 10.1158/1078-0432.CCR-13-2830

[19] Rudensky AY . Regulatory T cells and Foxp3. Immunol Rev 2011; 241: 260–268. 21488902 10.1111/j.1600-065X.2011.01018.x PMC3077798

[20] Sakaguchi S , Yamaguchi T , Nomura T , Ono M . Regulatory T cells and immune tolerance. Cell 2008; 133: 775–787. 18510923 10.1016/j.cell.2008.05.009

[21] Salama P , Phillips M , Grieu F *et al* Tumor‐infiltrating FOXP3^+^ T regulatory cells show strong prognostic significance in colorectal cancer. J Clin Oncol 2009; 27: 186–192. 19064967 10.1200/JCO.2008.18.7229

[22] Saito T , Nishikawa H , Wada H *et al* Two FOXP3^+^CD4^+^ T cell subpopulations distinctly control the prognosis of colorectal cancers. Nat Med 2016; 22: 679–684. 27111280 10.1038/nm.4086

[23] Miyara M , Yoshioka Y , Kitoh A *et al* Functional delineation and differentiation dynamics of human CD4^+^ T cells expressing the FoxP3 transcription factor. Immunity 2009; 30: 899–911. 19464196 10.1016/j.immuni.2009.03.019

[24] Feichtenbeiner A , Haas M , Buttner M , Grabenbauer GG , Fietkau R , Distel LV . Critical role of spatial interaction between CD8^+^ and Foxp3^+^ cells in human gastric cancer: the distance matters. Cancer Immunol Immunother 2014; 63: 111–119. 24170095 10.1007/s00262-013-1491-x PMC11029441

[25] Posselt R , Erlenbach‐Wunsch K , Haas M *et al* Spatial distribution of FoxP3^+^ and CD8^+^ tumour infiltrating T cells reflects their functional activity. Oncotarget 2016; 7: 60383–60394. 27494875 10.18632/oncotarget.11039 PMC5312390

[26] Szasz AM , Lanczky A , Nagy A *et al* Cross‐validation of survival associated biomarkers in gastric cancer using transcriptomic data of 1,065 patients. Oncotarget 2016; 7: 49322–49333. 27384994 10.18632/oncotarget.10337 PMC5226511

[27] Ayers M , Lunceford J , Nebozhyn M *et al* IFN‐γ–related mRNA profile predicts clinical response to PD‐1 blockade. J Clin Investig 2017; 127: 2930–2940. 28650338 10.1172/JCI91190 PMC5531419

[28] Hung K , Hayashi R , Lafond‐Walker A , Lowenstein C , Pardoll D , Levitsky H . The central role of CD4^+^ T cells in the antitumor immune response. J Exp Med 1998; 188: 2357–2368. 9858522 10.1084/jem.188.12.2357 PMC2212434

[29] Gacerez AT , Sentman CL . T‐bet promotes potent antitumor activity of CD4^+^ CAR T cells. Cancer Gene Ther 2018; 25: 117–128. 29515240 10.1038/s41417-018-0012-7 PMC6021366

[30] Dong H , Zhu G , Tamada K , Chen L . B7–H1, a third member of the B7 family, co‐stimulates T‐cell proliferation and interleukin‐10 secretion. Nat Med 1999; 5: 1365–1369. 10581077 10.1038/70932

[31] Fontenot JD , Gavin MA , Rudensky AY . Foxp3 programs the development and function of CD4^+^CD25^+^ regulatory T cells. Nat Immunol 2003; 4: 330–336. 12612578 10.1038/ni904

[32] Shevach EM . From vanilla to 28 flavors: multiple varieties of T regulatory cells. Immunity 2006; 25: 195–201. 16920638 10.1016/j.immuni.2006.08.003

[33] Williams LM , Rudensky AY . Maintenance of the Foxp3‐dependent developmental program in mature regulatory T cells requires continued expression of Foxp3. Nat Immunol 2007; 8: 277–284. 17220892 10.1038/ni1437

[34] deLeeuw RJ , Kost SE , Kakal JA , Nelson BH . The prognostic value of FoxP3^+^ tumor‐infiltrating lymphocytes in cancer: a critical review of the literature. Clin Cancer Res 2012; 18: 3022–3029. 22510350 10.1158/1078-0432.CCR-11-3216

[35] Curiel TJ , Coukos G , Zou L *et al* Specific recruitment of regulatory T cells in ovarian carcinoma fosters immune privilege and predicts reduced survival. Nat Med 2004; 10: 942–949. 15322536 10.1038/nm1093

[36] Siddiqui SA , Frigola X , Bonne‐Annee S *et al* Tumor‐infiltrating Foxp3^‐^CD4^+^CD25^+^ T cells predict poor survival in renal cell carcinoma. Clin Cancer Res 2007; 13: 2075–2081. 17404089 10.1158/1078-0432.CCR-06-2139

[37] Carreras J , Lopez‐Guillermo A , Fox BC *et al* High numbers of tumor‐infiltrating FOXP3‐positive regulatory T cells are associated with improved overall survival in follicular lymphoma. Blood 2006; 108: 2957–2964. 16825494 10.1182/blood-2006-04-018218

[38] Fridman WH , Zitvogel L , Sautes‐Fridman C , Kroemer G . The immune contexture in cancer prognosis and treatment. Nat Rev Clin Oncol 2017; 14: 717–734. 28741618 10.1038/nrclinonc.2017.101

[39] Association of response to programmed death receptor 1 (PD‐1) blockade with pembrolizumab (MK‐3475) with an interferon‐inflammatory immune gene signature. *Proc Am Soc Clin Onol* 2015.

[40] Ayers M , Lunceford J , Nebozhyn M *et al* IFN‐γ‐related mRNA profile predicts clinical response to PD‐1 blockade. J Clin Invest 2017; 127: 2930–2940. 28650338 10.1172/JCI91190 PMC5531419

[41] Pattison S , Mann GB , Crosthwaite G *et al* Predictors of outcome after surgery for gastric cancer in a Western cohort. ANZ J Surg 2016; 86: 469–474. 25388659 10.1111/ans.12915

[42] Ying L , Yan F , Meng Q *et al* PD‐L1 expression is a prognostic factor in subgroups of gastric cancer patients stratified according to their levels of CD8 and FOXP3 immune markers. Oncoimmunology 2018; 7: e1433520. 29872566 10.1080/2162402X.2018.1433520 PMC5980489

[43] Huang YK , Wang M , Sun Y *et al* Macrophage spatial heterogeneity in gastric cancer defined by multiplex immunohistochemistry. Nat Commun 2019; 10: 3928. 31477692 10.1038/s41467-019-11788-4 PMC6718690

[44] Busuttil RA , George J , Tothill RW *et al* A signature predicting poor prognosis in gastric and ovarian cancer represents a coordinated macrophage and stromal response. Clin Cancer Res 2014; 20: 2761–2772. 24658156 10.1158/1078-0432.CCR-13-3049

[45] Ritchie ME , Phipson B , Wu D *et al* limma powers differential expression analyses for RNA‐sequencing and microarray studies. Nucleic Acids Res 2015; 43: e47. 25605792 10.1093/nar/gkv007 PMC4402510

[46] Bindea G , Mlecnik B , Hackl H *et al* ClueGO: a Cytoscape plug‐in to decipher functionally grouped gene ontology and pathway annotation networks. Bioinformatics 2009; 25: 1091–1093. 19237447 10.1093/bioinformatics/btp101 PMC2666812

[47] Bindea G , Galon J , Mlecnik B . CluePedia Cytoscape plugin: pathway insights using integrated experimental and in silico data. Bioinformatics 2013; 29: 661–663. 23325622 10.1093/bioinformatics/btt019 PMC3582273

[48] Shannon P , Markiel A , Ozier O *et al* Cytoscape: a software environment for integrated models of biomolecular interaction networks. Genome Res 2003; 13: 2498–2504. 14597658 10.1101/gr.1239303 PMC403769

[49] Finck R , Simonds EF , Jager A *et al* Normalization of mass cytometry data with bead standards. Cytometry A 2013; 83: 483–494. 23512433 10.1002/cyto.a.22271 PMC3688049

[50] Chen TJ , Kotecha N . Cytobank: providing an analytics platform for community cytometry data analysis and collaboration. Curr Top Microbiol Immunol 2014; 377: 127–157. 24590675 10.1007/82_2014_364

[51] Diggins KE , Ferrell PB Jr , Irish JM . Methods for discovery and characterization of cell subsets in high dimensional mass cytometry data. Methods 2015; 82: 55–63. 25979346 10.1016/j.ymeth.2015.05.008 PMC4468028

