# Supplementary figures and images for: High‐dimensional analyses reveal a distinct role of T‐cell subsets in the immune microenvironment of gastric cancer
Source: Clin Transl Immunology. 2020 May 5;9(5):e1127. doi: 10.1002/cti2.1127 (PMC7200219; doi:10.1002/cti2.1127)

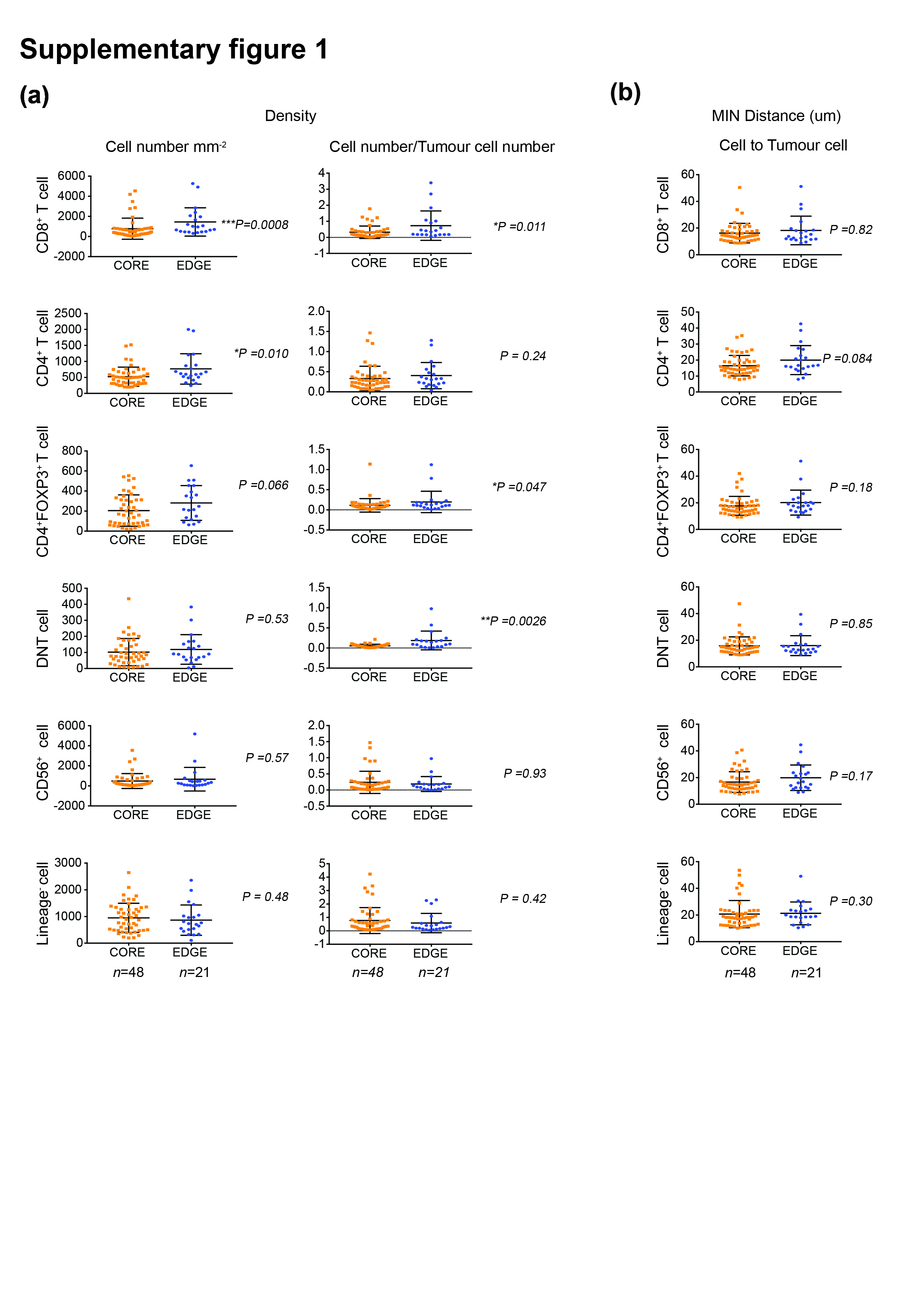

Supplement: Supplementary file 1 [file CTI2-9-e1127-s001.jpg]

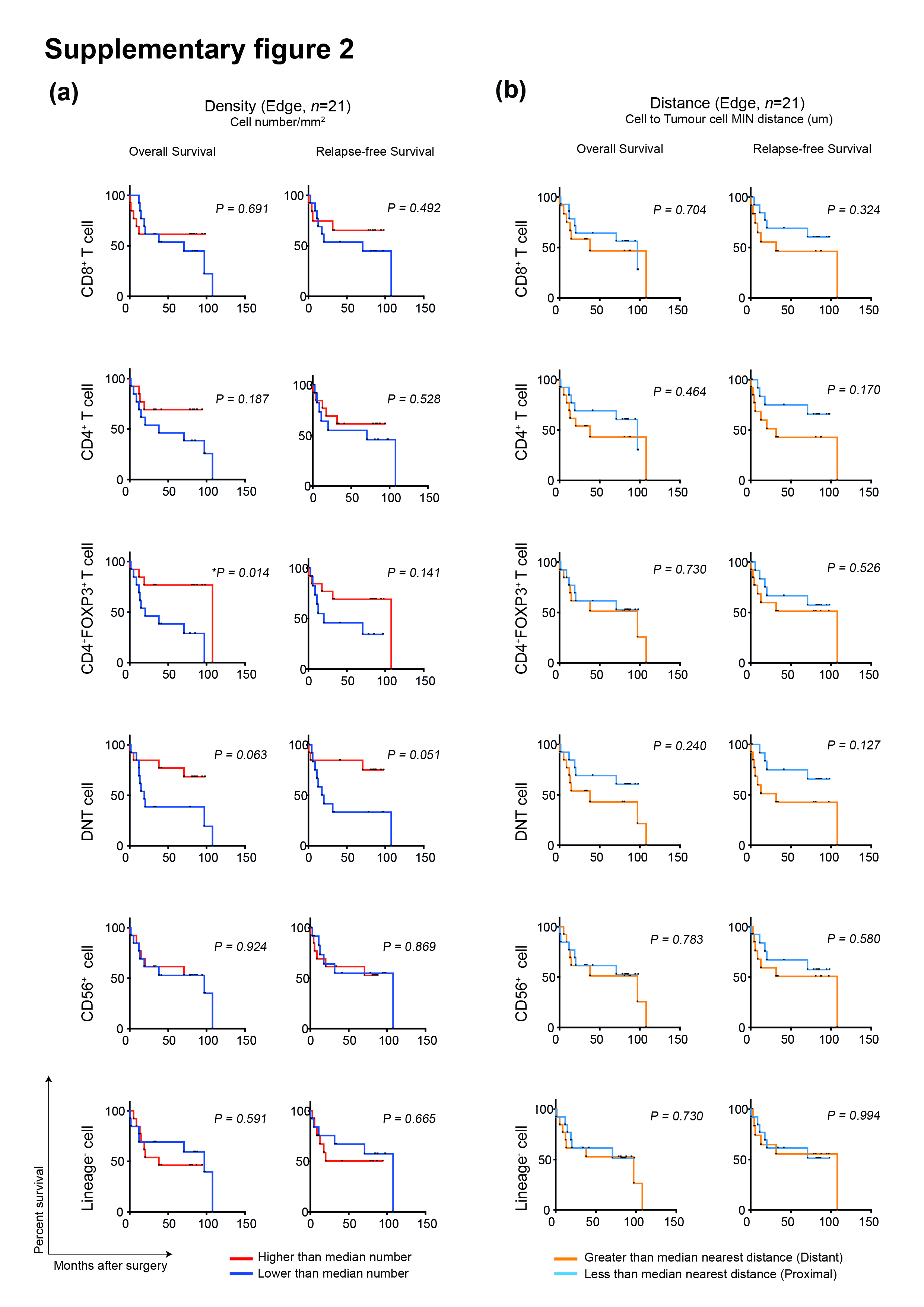

Supplement: Supplementary file 2 [file CTI2-9-e1127-s002.jpg]

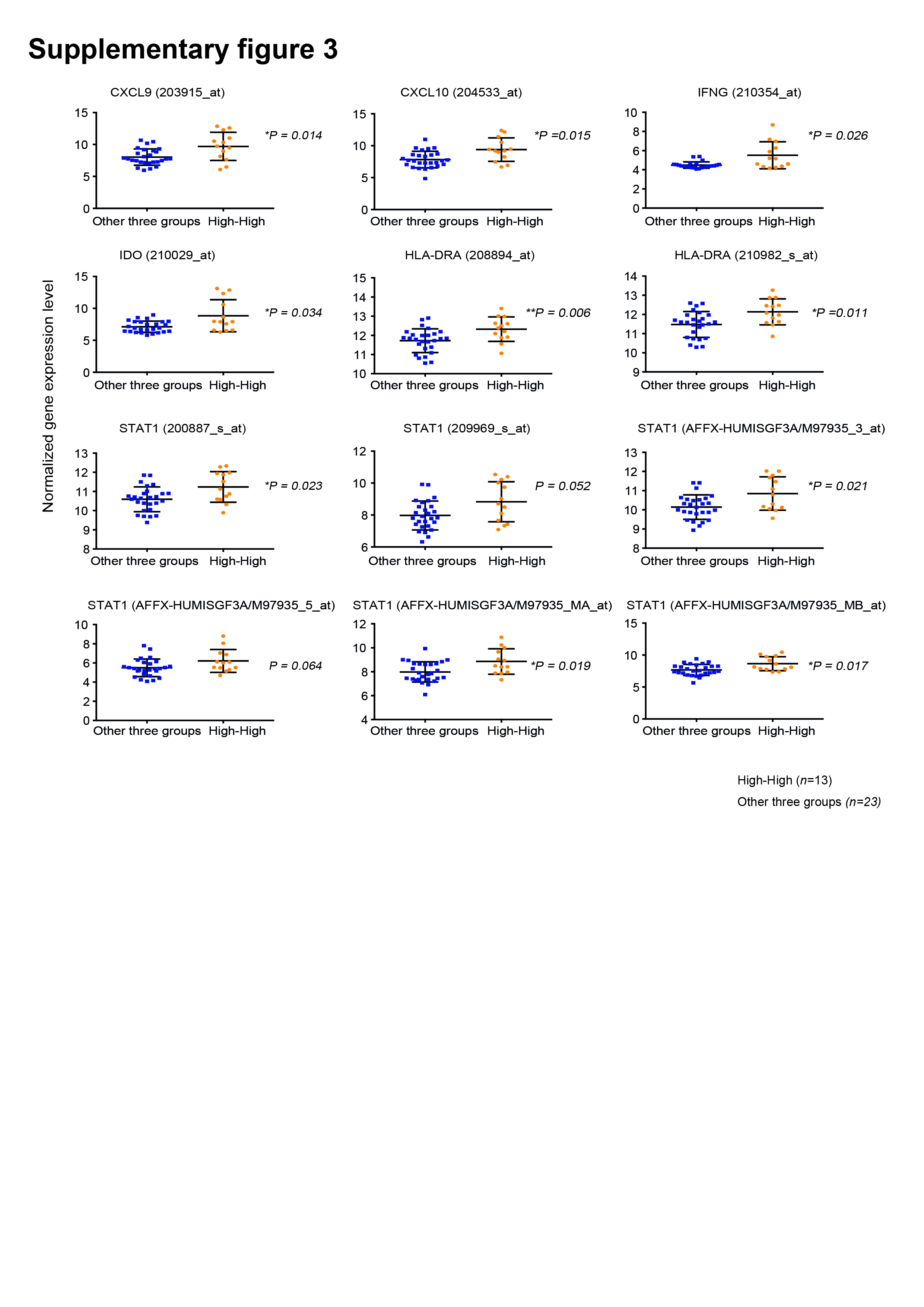

Supplement: Supplementary file 3 [file CTI2-9-e1127-s003.jpg]

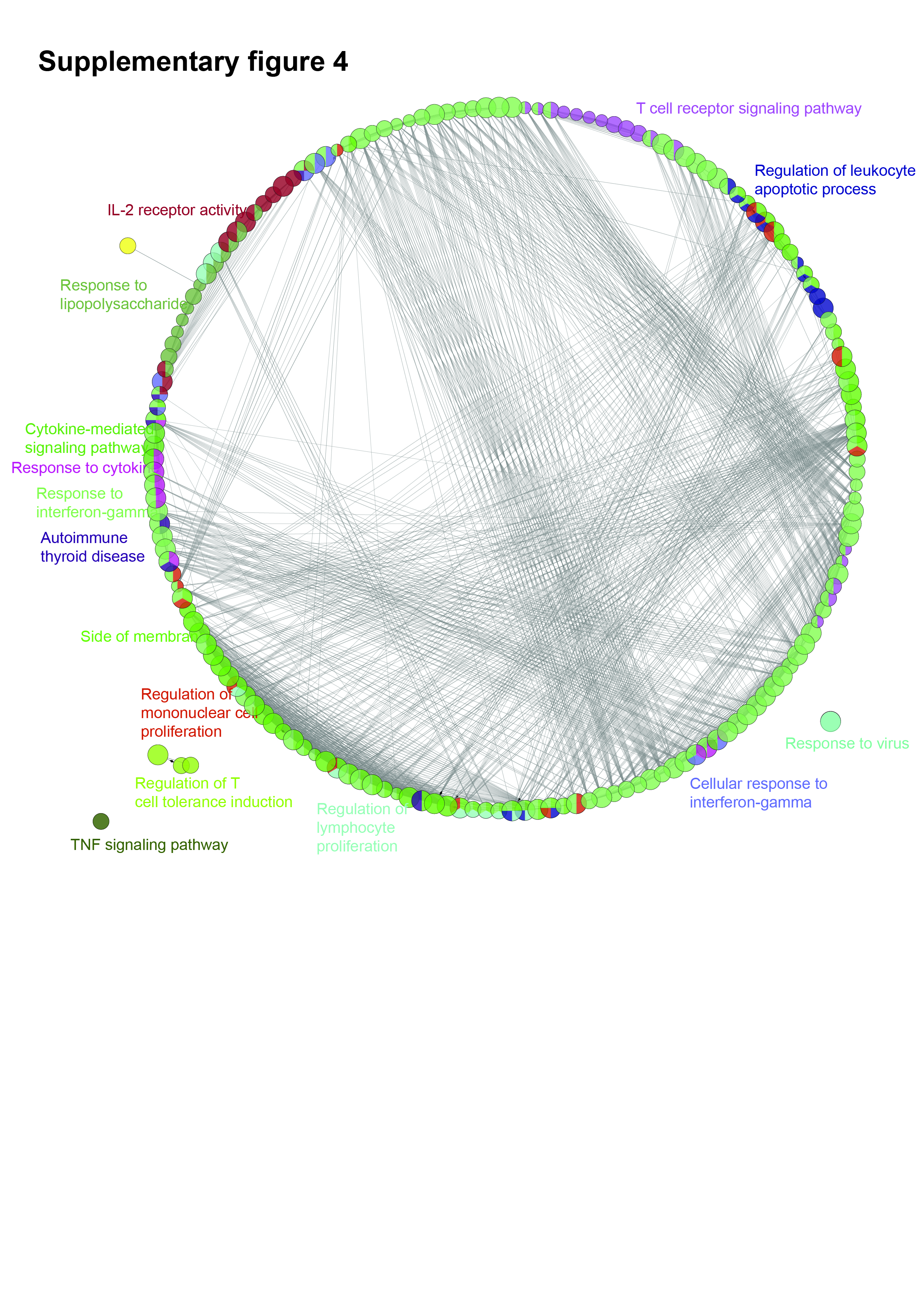

Supplement: Supplementary file 4 [file CTI2-9-e1127-s004.jpg]

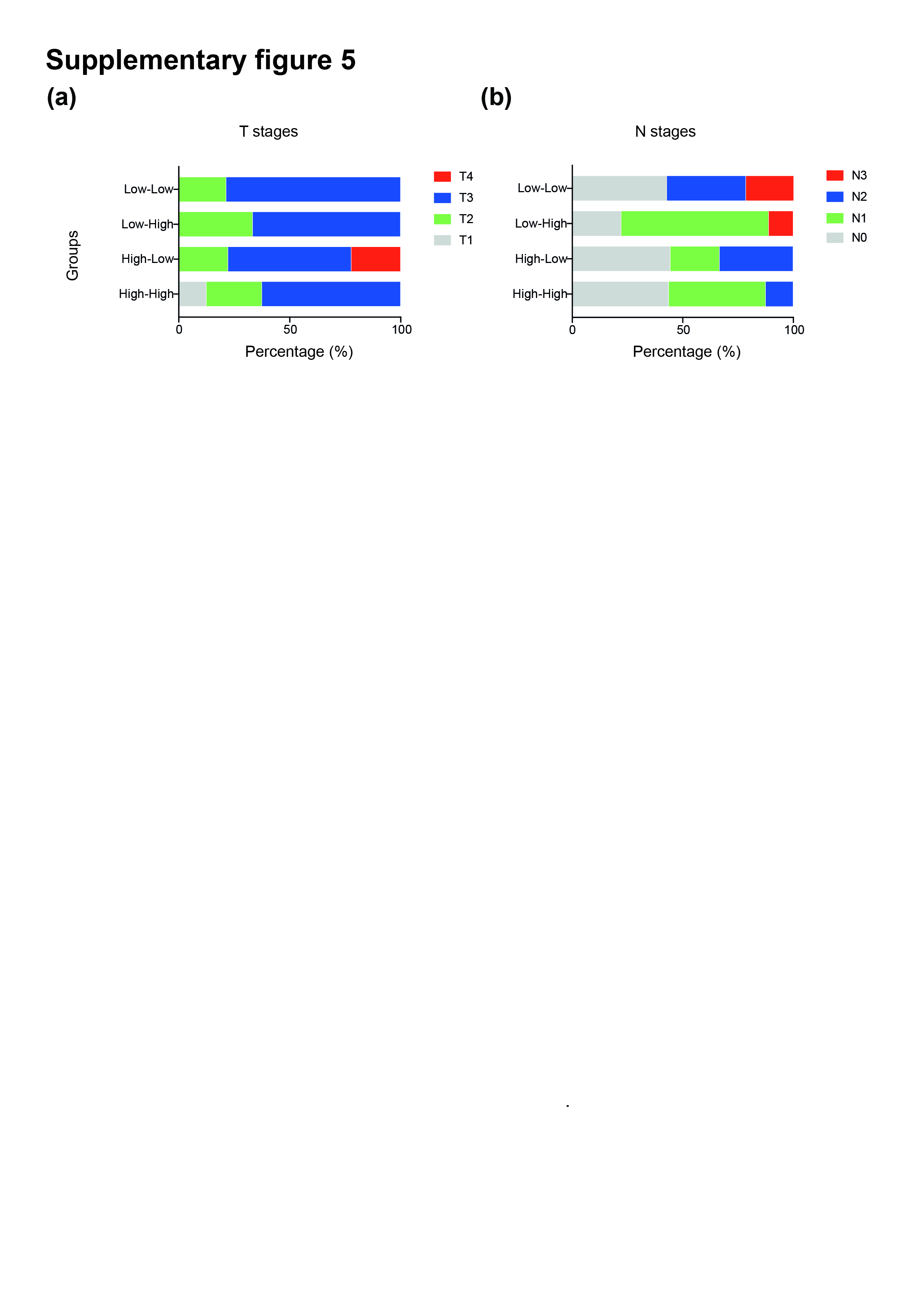

Supplement: Supplementary file 5 [file CTI2-9-e1127-s005.jpg]

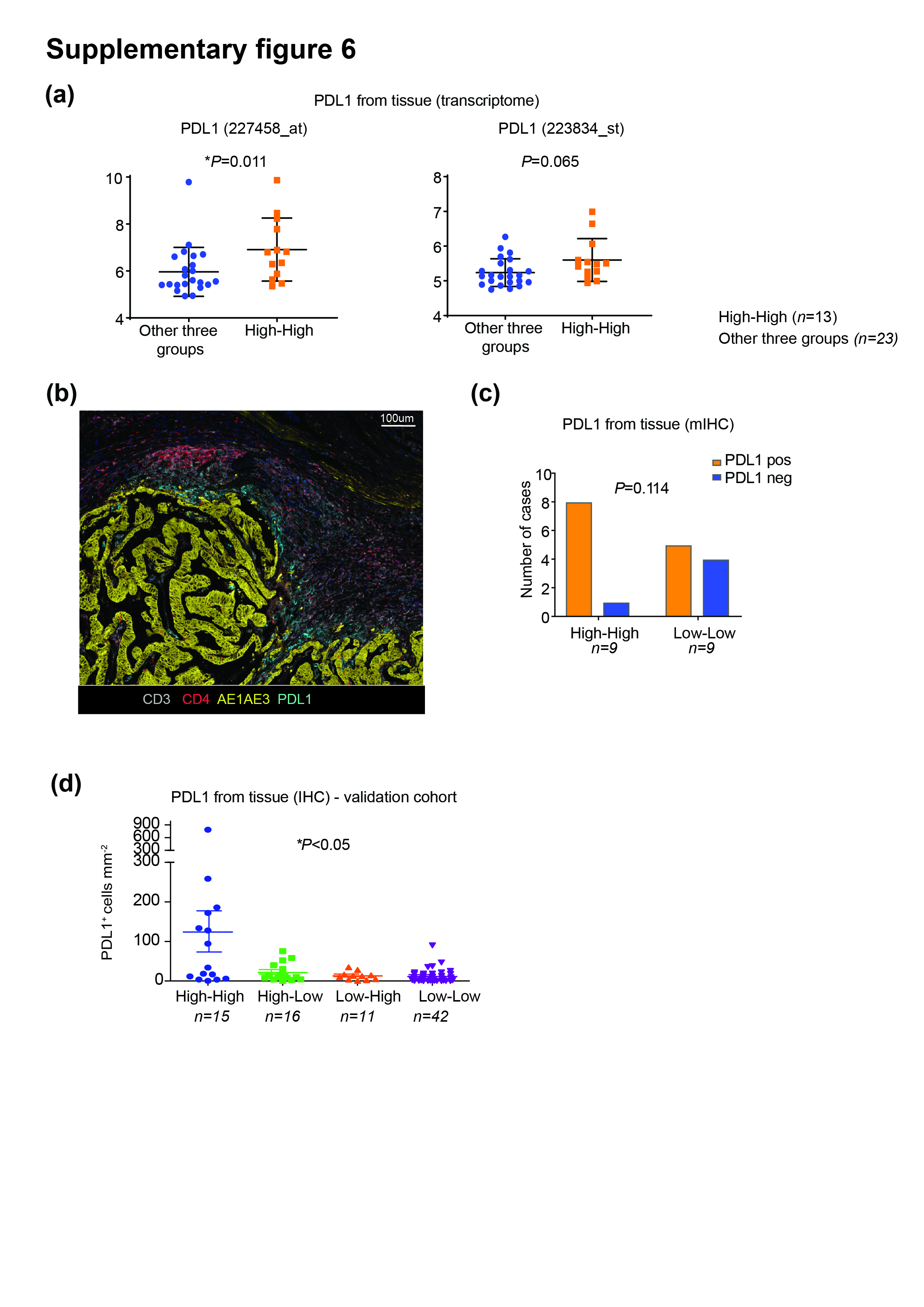

Supplement: Supplementary file 6 [file CTI2-9-e1127-s006.jpg]

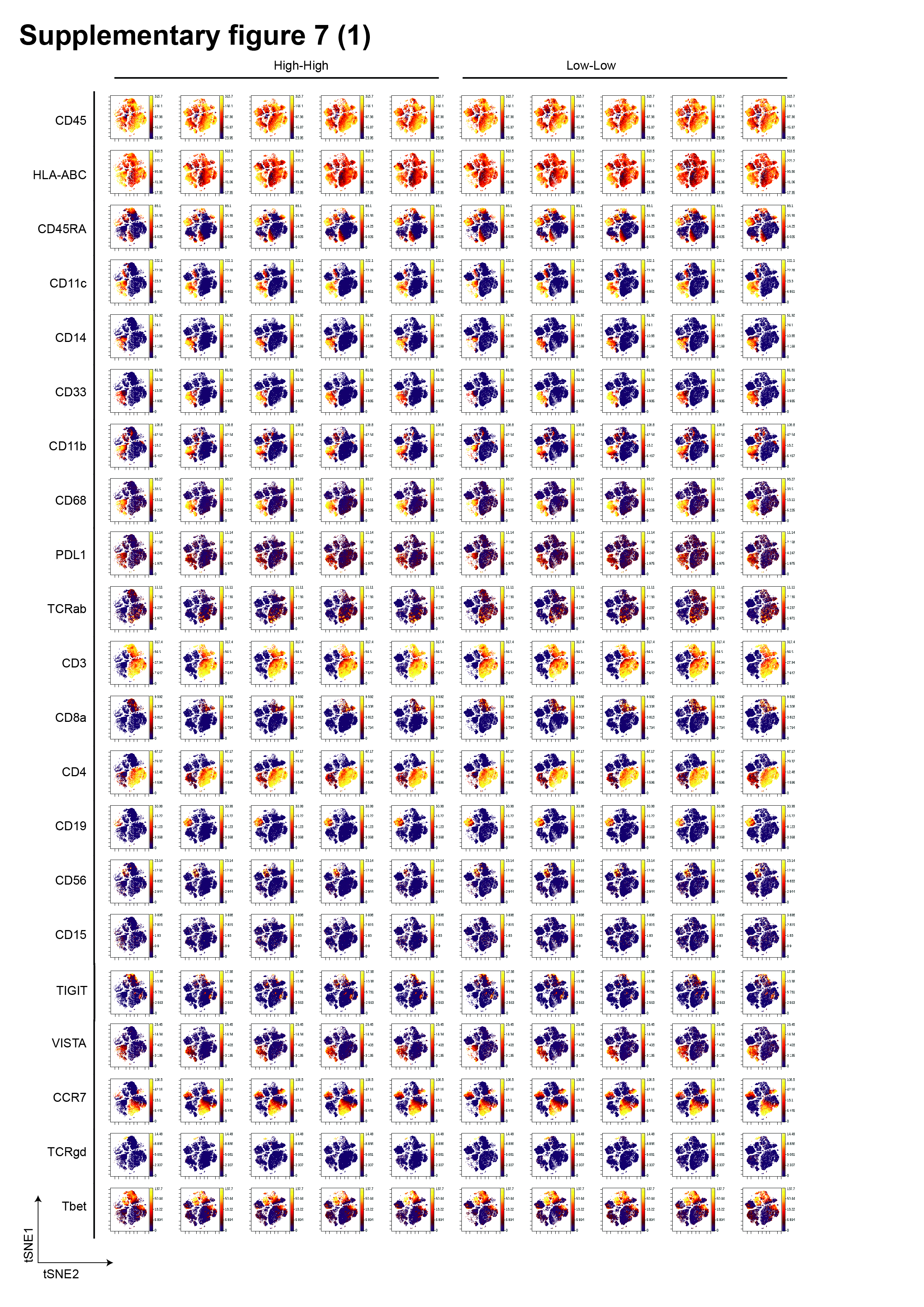

Supplement: Supplementary file 7 [file CTI2-9-e1127-s007.jpg]

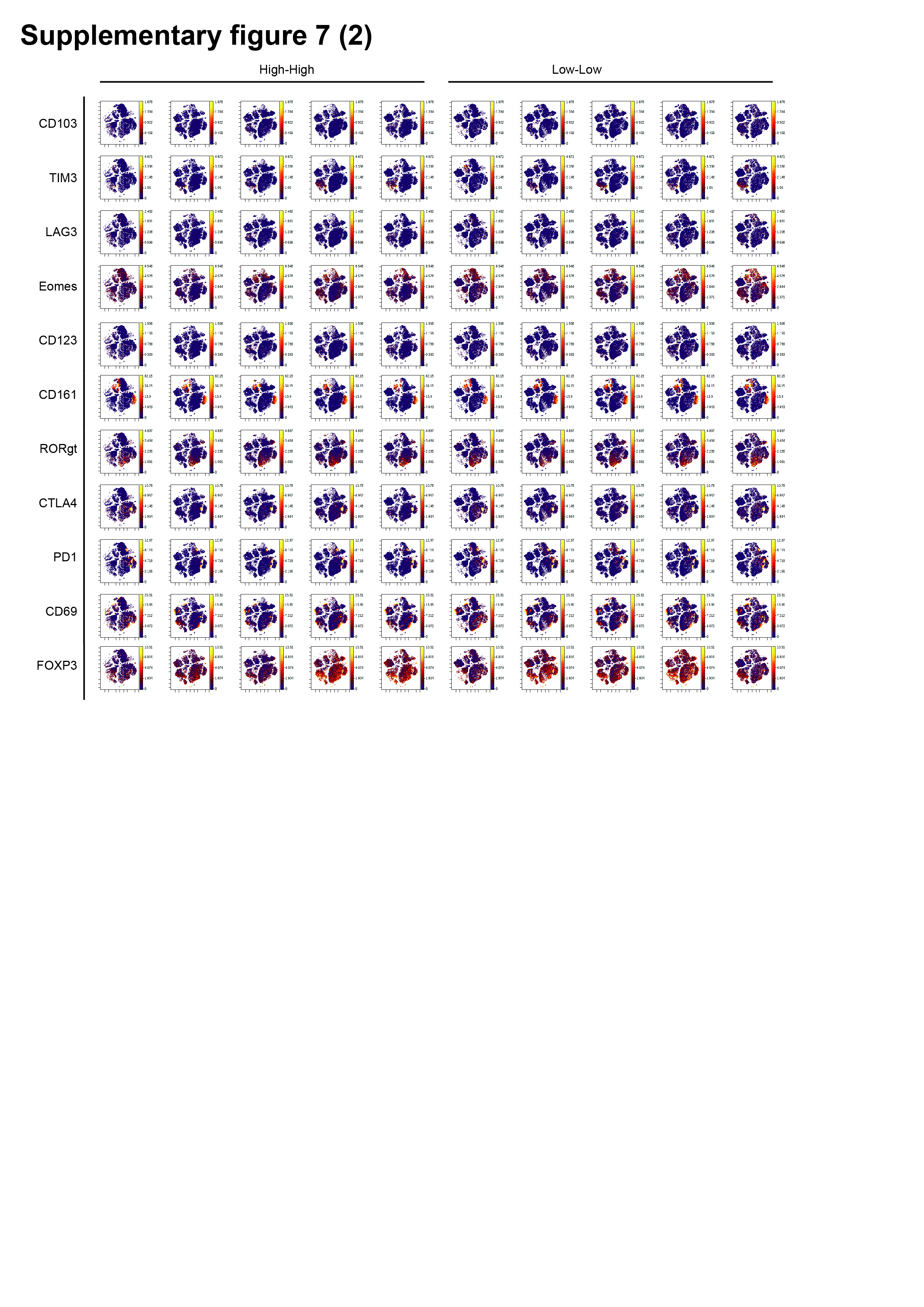

Supplement: Supplementary file 8 [file CTI2-9-e1127-s008.jpg]

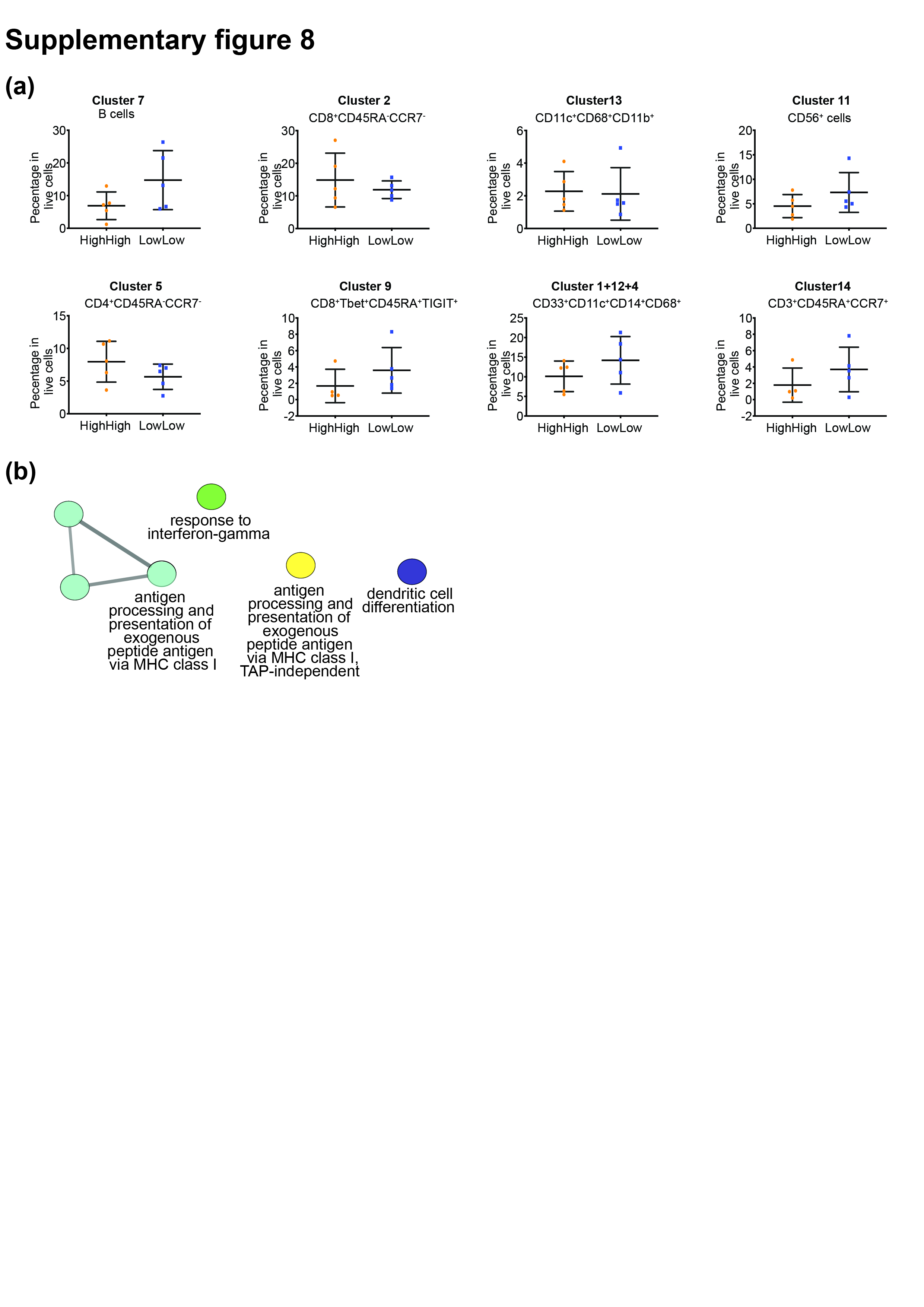

Supplement: Supplementary file 9 — Supplementary figures 1‐5 [file CTI2-9-e1127-s009.jpg]
