# Supplementary material for: High‐dimensional analyses reveal a distinct role of T‐cell subsets in the immune microenvironment of gastric cancer
Source: Clin Transl Immunology. 2020 May 5;9(5):e1127. doi: 10.1002/cti2.1127 (PMC7200219; doi:10.1002/cti2.1127)
Supplement: Supplementary file 10 — Supplementary tables 1‐5 [file CTI2-9-e1127-s010.docx]

## Supplementary tables

Supplementary table 1. Multivariate Cox regression analysis for survival among GC patients

| Variable | Overall survival (OS) | | | |
| --- | --- | --- | --- | --- |
|  | HR | B | SE | *P-*value |
| **Age**  10 years interval | 1.498 | 0.404 | 0.173 | 0.020 |
| **AJCC stage**  I to III | 1.952 | 0.669 | 0.207 | 0.001 |
| **CD4^+^FOXP3^+^ T cell number**  High *vs*. Low | 0.238 | -1.435 | 0.404 | <0.001 |
| **CD4^+^FOXP3^+^ T cell to tumour cell MIN distance**  Distant *vs* Proximal | 2.469 | 0.904 | 0.378 | 0.017 |

Supplementary table 2. Data summary in the Kaplan Meier survival curves for the duration of OS and RFS according to the numbers of CD8 T cells and CD4+FOXP3+ T cells

|  | CD8 + CD4FOXP3 | | | |
| --- | --- | --- | --- | --- |
|  | Low-Low | High-Low | Low-High | High-High |
| n | 14 | 9 | 9 | 16 |
| OS |  |  |  |  |
| # censored subjects | 1 | 1 | 3 | 9 |
| # deaths | 13 | 8 | 6 | 7 |
| Median survival (months) | 16.32 | 15.90 | 27.77 | 97.03 |
| RFS |  |  |  |  |
| # censored subjects | 2 | 4 | 3 | 11 |
| # recurrence | 12 | 5 | 6 | 5 |
| Median survival (months) | 14.03 | 24.87 | 20.60 | Undefined |

Supplementary table 3. Multivariable Cox proportional hazard analysis for OS and RFS among GC patients

| **Viable** | ***n* (%)** | **Overall Survival (OS)** | | **Relapse-free Survival (RFS)** | |
| --- | --- | --- | --- | --- | --- |
|  |  | **HR (95% CI)** | ***P*-value** | **HR (95% CI)** | ***P*-value** |
| **Unadjusted Cox model** 0.10 0.10 | | | | | |
| **AJCC stage (7th)** | |  |  |  |  |
| Stage I+II | 29 (60.42%) | 0.55 (0.28-1.10) | 0.093 | 0.57 (0.27-1.20) | 0.14 |
| Stage III | 19 (39.58%) | 1.0 (reference) |  | 1.0 (reference) |  |
| **Lauren classification** | |  | 0.02* |  | 0.06 |
| Diffuse | 14 (29.17%) | 1.0 (reference) |  | 1.0 (reference) |  |
| Intestinal | 30 (62.50%) | 0.38 (0.18-0.81) | 0.011* | 0.48 (0.21-1.09) | 0.080 |
| Mixed | 4 (8.33%) | 1.38 (0.44-4.31) | 0.574 | 1.74 (0.54-5.67) | 0.356 |
| **Molecular subtypes** | |  | 0.03* |  | 0.03* |
| EBV/MSI | 9 (18.75%) | 1.0 (reference) |  | 1.0 (reference) |  |
| GS/CIN | 39 (81.25%) | 3.08 (0.94-10.17) | 0.064 | 3.79 (0.89-16.08) | 0.070 |
| **CD8+CD4FOXP3** | |  | 0.002** |  | 0.007** |
| High-High | 16 (33.33%) | 1.0 (reference) |  | 1.0 (reference) |  |
| Other three groups | 32 (66.67%) | 3.63 (1.48-8.87) | 0.005** | 3.36 (1.27-8.94) | 0.015* |
| **Multivariable Cox Model** 0.002* 0.02*  **with AJCC stage, Lauren classification, Molecular subtypes and CD8+CD4FOXP3** | | | | | |
| **AJCC stage (7th)** | |  |  |  |  |
| Stage I+II | 29 (60.42%) | 0.40 (0.19-0.84) | 0.016* | 0.47 (0.22-1.02) | 0.06 |
| Stage III | 19 (39.58%) | 1.0 (reference) |  | 1.0 (reference) |  |
| **Lauren classification** | |  |  |  |  |
| Diffuse | 14 (29.17%) | 1.0 (reference) |  | 1.0 (reference) |  |
| Intestinal | 30 (62.50%) | 0.60 (0.26-1.39) | 0.24 | 0.84 (0.34-2.08) | 0.70 |
| Mixed | 4 (8.33%) | 1.46 (0.47-4.58) | 0.51 | 1.80 (0.55-5.87) | 0.33 |
| **Molecular subtypes** | |  |  |  |  |
| EBV/MSI | 9 (18.75%) | 1.0 (reference) |  | 1.0 (reference) |  |
| GS/CIN | 39 (81.25%) | 1.63 (0.45-5.95) | 0.46 | 2.18 (0.46-10.34) | 0.33 |
| **CD8+CD4FOXP3** | |  |  |  |  |
| High-High | 16 (33.33%) | 1.0 (reference) |  | 1.0 (reference) |  |
| Other three groups | 32 (66.67%) | 2.86 (0.98-8.29) | 0.05* | 2.58 (0.82-8.21) | 0.11 |

*AJCC 7th: American Joint Committee on Cancer staging system, 7th edition; EBV: Epstein-Barr virus; MSI: microsatellite instability; GS: genomically stable; CIN: chromosomal instability; HR: hazard ratio; CI: confidence interval.

Supplementary table 4. Sequential multiplex IHC staining protocol

| Cell Marker (Host) | Primary Ab Company (Clone) | Dilutions | Secondary Ab (Dilutions) | TSA Plus (Dilutions) | Color-applied |
| --- | --- | --- | --- | --- | --- |
| CD3 | Spring Bioscience (SP7) | 1:500 | Anti-rabbit IgG (1:1000) | 520 (1:50) | Grey |
| CD8 | Thermo Fisher (4B11) | 1:100 | Anti-mouse IgG (1:500) | 620 (1:50) | Green |
| CD4 | Spring Bioscience (SP35) | 1:100 | Anti-rabbit IgG (1:1000) | 650 (1:50) | Red |
| FOXP3 | BioSB (polyclonal) | 1:100 | Anti-rabbit IgG (1:1000) | 570 (1:50) | Orange |
| CD56 | Leica (CD564) | 1:100 | Anti-mouse IgG (1:250) | 540 (1:50) | Yellow |
| PDL1 | Spring Bioscience (SP142) | 1:1000 | Anti-Rabbit IgG (1:1000) | 540 (1:50) | Cyan |
| AE1AE3 | Leica (AE1 and AE3 cocktail) | 1:200 | Anti-mouse IgG (1:500) | 690 (1:50) | Magenta |

Supplementary table 5. List of antibodies for CyTOF

| Metal tag | Marker | Clone | Source | Staining |
| --- | --- | --- | --- | --- |
| 89Y | CD45 | H130 | Fluidigm | Surface markers |
| 115In | CD8a | RPA-T8 | Biolegend |  |
| 142Nd | CD19 | HIB19 | Biolegend |  |
| 143Nd | CD123 | 6H6 | Biolegend |  |
| 144Nd | HLA-ABC | W6-32 | Fluidigm |  |
| 145Nd | CD4 | RPA-T4 | Biolegend |  |
| 147Sm | CD11c | Bu15 | Biolegend |  |
| 148Nd | CD274 (PDL1) | 29E.2A3 | Biolegend |  |
| 151Eu | CD103 | Ber-ACT8 | Fluidigm |  |
| 152Sm | TCRgd | 11F2 | Fluidigm |  |
| 154Sm | CD45RA | HI100 | Biolegend |  |
| 155Gd | TIGIT | A15153G | Biolegend |  |
| 157Gd | TIM3 | F38-2E2 | Biolegend |  |
| 158Gd | CD33 | WM53 | Fluidigm |  |
| 159Tb | CD161 | HP-3G16 | Fluidigm |  |
| 161Dy | CD69 | FN50 | Biolegend |  |
| 163Dy | CD56 | HCD56 | Biolegend |  |
| 164Dy | CD15 | W6D3 | Fluidigm |  |
| 165Ho | LAG3 | 11C3C65 | Biolegend |  |
| 167Er | CCR7 | G043H7 | Biolegend |  |
| 170Rt | CD3 | UCHT1 | Biolegend |  |
| 173Yb | VISTA | 73O8O4 | R&D Systems |  |
| 174Yb | CD279 (PD1) | EH12.2H7 | Biolegend |  |
| 175Lu | CD14 | M5E2 | Biolegend |  |
| 176Yb | TCRab | IP26 | Biolegend |  |
| 209Bi | CD11b | ICRF44 | Fluidigm |  |
| 153Eu | Eomes | WD1928 | Invitrogen | Intercellular markers |
| 160Gd | Tbet | 4B10 | Fluidigm |  |
| 162Dy | Foxp3 | PCH101 | Fluidigm |  |
| 168Er | RORgt | 1181A | R&D Systems |  |
| 169Tm | CTLA4 | BN13 | Biolegend |  |
| 171Yb | CD68 | Y1/82A | Fluidigm |  |

Antibodies not purchased from Fluidigm were purchased in purified form from the listed sources, and metal conjugated in house using X8 Multi-Metal Labelling Kit (Fluidigm).

## Supplementary figure legends

Supplementary figure 1. Heterogeneous immune cell distribution between tumour core and tumour edge in the gastric cancer tumour microenvironment.

(a) Immune subset cells were counted and represented as tissue density (cells per mm2) and tissue density as a ratio with tumour cells. Densities were compared between tumour core (*n*=48) and tumour edge (*n*=21) for CD8^+^ T cells, CD4^+^ T cells, CD4^+^FOXP3^+^ T cells, DNT cells, CD56^+^ cells and Lineage^-^ cells. (b) The ‘median intercellular nearest’ (MIN) distance to tumour cells were compared between tumour core (*n*=48) and tumour edge (*n*=21) for CD8^+^ T cells, CD4^+^ T cells, CD4^+^FOXP3^+^ T cells, DNT cells, CD56^+^ cells and Lineage^-^ cells. Data are presented as the mean ± SD. Significance was determined using the two-tailed Mann-Whitney *U*-test. * *P*<0.05, ** *P*<0. 01, *** *P*<0. 001. DNT cell: Double negative T cell.

Supplementary figure 2. Association of densities and spatial distributions of immune cells subsets from tumour edge (*n*=21) with patients’ survival.

(**a**) Overall survival (OS; left) and relapse-free survival (RFS; right) analyses based on densities of CD8^+^ T cells, CD4^+^ T cells, CD4^+^FOXP3^+^ T cells, DN T cells, CD56^+^ cells, and Lineage**^-^** cells from tumour edge (*n*=21). Individual immune infiltrate values were divided into higher or lower based on the median number of cells. (**b**) OS (left) and RFS (right) analyses based on MIN distance to tumour cells from CD8^+^ T cells, CD4^+^ T cells, CD4^+^FOXP3^+^ T cells, DN T cells, CD56^+^ cells, and Lineage**^-^** cells from tumour edge (*n*=21). Distant and proximal distance values were defined based on the MIN distance. Hazard ratio (HR) was shown for lower compared to higher for density analysis, and distant compared to proximal for distance analysis. HR and 95% confidence interval are shown. Significance was determined using the log-rank Mantel-Cox test. * *P*<0.05, ** *P*<0. 01, *** *P*<0. 001. DNT cell: Double negative T cell.

Supplementary figure 3. Individual probe sets of IFN-γ related genes (CXCL9, CXCL10, IDO1, IFNG, HLA-DRA, and STAT1) were significantly up-regulated in the High-High group patients compared to the other three groups.

Data are presented as the mean ± SD. Significance was determined using the two-tailed Mann-Whitney *U*-test. *P-*value<0.05 were considered statistically significant * *P*<0.05, ** *P*<0. 01, *** *P*<0. 001. DNT cell: Double negative T cell.

Supplementary figure 4. Enriched pathways in the High-High group.

Nodes represent enriched pathways based on shared genes and linked based on the k score in network format. Shown with pathways with *P*-value <0.01.

Supplementary figure 5. Tumour depths (T stages) and affected lymph nodes (N stage) distribution in four groups at the time of surgery.

(**a**) Tumour depths (T stages) proportion distribution in High-High, High-Low, Low-High, and Low-Low groups at the time of surgery. (**b**) Affected lymph nodes (N stages) proportion distribution in High-High, High-Low, Low-High, and Low-Low groups at the time of surgery.

Supplementary figure 6. PDL1 upregulation in the High-High GC tumours.

(**a)** PDL1 over-expressed in the tumour tissue from the High-High group using microarray data (*n*=36). Significance was determined using the two-tailed Mann-Whitney *U*-test. (**b)** Representative image of CD3, CD4 and PDL1 staining on gastric tumour tissue. (**c**) The High-High group were enriched with PDL1 positive cases determined by mIHC (*n*=18). Significance was determined using the two-sided Chi-square test (**d**) PDL1 positive cells in the High-High group from the validation cohort compared to the other three groups using mIHC (*n*=84). Significance was determined using Kruskal-Walis test. Data are presented as the mean ± SD. * *P*<0.05, ** *P*<0. 01, *** *P*<0. 001.

Supplementary figure 7. Visualisation grid of viSNE plots with 36 measured parameters.

Visualisation grid of viSNE plots with plots arranged according to marker expression (rows) relative to individuals (columns) are shown. Data show all viable single cells, subjected to the tSNE algorithm, which provides each cell with a unique coordinate according to its expression of the 36 measured parameters, displayed on a two-dimensional plot (tSNE1 and tSNE2).

Supplementary figure 8. The High-High GS/CIN tumours were enriched for genes active in antigen processing and presentation, IFN-γ response, and DC differentiation.

(**a**) Other clusters, including B cells (C7), CD56^+^ cells(C11), CD8^+^ cells (C2 and C9), CD4^+^CCR7^-^CD45RA^-^ cells (C5) and monocytes (C1, C4, and C12) did not show significant differences between the two groups. Data are presented as the mean ± SD. Significance was determined using the two-tailed Mann-Whitney *U*-test. (**b**) Pathways enriched in the High-High GS/CIN tumours compared to other GS/CIN tumour. Shown with pathways with *P*-value <0.01. GS: genomically stable; CIN: chromosomal instability.
